# B-type natriuretic peptide is upregulated by c-Jun N-terminal kinase and contributes to septic hypotension

**DOI:** 10.1172/jci.insight.133675

**Published:** 2020-04-23

**Authors:** Matthew Hoffman, Ioannis D. Kyriazis, Alexandra Dimitriou, Santosh K. Mishra, Walter J. Koch, Konstantinos Drosatos

**Affiliations:** 1 Center for Translational Medicine, Temple University Lewis Katz School of Medicine, Philadelphia, Pennsylvania, USA.; 2 Department of Molecular Biomedical Sciences, North Carolina State University, Raleigh, North Carolina, USA.; 3 Center for Metabolic Disease Research, Temple University Lewis Katz School of Medicine, Philadelphia, Pennsylvania, USA.

**Keywords:** Cardiology, Heart failure, Mouse models, Signal transduction

## Abstract

B-type natriuretic peptide (BNP) is secreted by ventricular cardiomyocytes in response to various types of cardiac stress and has been used as a heart failure marker. In septic patients, increased BNP suggests poor prognosis; however, no causal link has been established. Among various effects, BNP decreases systemic vascular resistance and increases natriuresis that leads to lower blood pressure. We previously observed that JNK inhibition corrects cardiac dysfunction and suppresses cardiac BNP mRNA in endotoxemia. In this study, we investigated the transcriptional mechanism that regulates BNP expression and the involvement of plasma BNP in causing septic hypotension. Our in vitro and in vivo findings confirmed that activation of JNK signaling increases BNP expression in sepsis via direct binding of c-Jun in activating protein–1 (AP-1) regulatory elements of the *Nppb* promoter. Accordingly, genetic ablation of BNP, as well as treatment with a potentially novel neutralizing anti-BNP monoclonal antibody (19B3) or suppression of its expression via administration of JNK inhibitor SP600125 improved cardiac output, stabilized blood pressure, and improved survival in mice with polymicrobial sepsis. Therefore, inhibition of JNK signaling or BNP in sepsis appears to stabilize blood pressure and improve survival.

## Introduction

Sepsis results from the overwhelming systemic response to infection resulting in hemodynamic instability, reduced tissue perfusion, and multiple organ dysfunction (1). Despite substantial effort put forth to understand the molecular mechanisms underlying sepsis resulting in numerous clinical trials, no targeted therapies have proven effective at improving patient care, and supportive treatment is the standard (2). Sepsis is diagnosed based on a sequential organ failure assessment (SOFA) score greater than 2 (3), and cardiovascular complications in sepsis increase mortality (4, 5). The systemic host response promotes cardiovascular dysfunction, resulting in increased vascular permeability, relative hypovolemia, and reduced arterial pressure. The resultant hypotension in sepsis dramatically increases risk of mortality, and refractory hypotension is diagnostic for the most severe category of sepsis, septic shock (3, 6, 7).

Circulating B-type natriuretic peptide (BNP) has been proposed as a biomarker associated with poor prognosis and myocardial depression resulting from severe sepsis (4, 5, 8, 9). However, no causal link has been described. BNP is encoded by the *Nppb* gene and is produced as a pre-pro-peptide by the ventricular myocytes in response to myocardial stress. In turn, BNP interacts with the guanylate cyclase–coupled natriuretic peptide receptor A (NPR-A) to reduce preload and afterload by promoting vasodilation, reducing venous return, reducing sympathetic outflow, and promoting natriuresis (10–12). Previously, we demonstrated — using a mouse model of polymicrobial sepsis (cecal ligation and puncture; CLP) — that rapid progression to a hypodynamic state is associated with increased plasma BNP levels within 2 hours of sepsis induction (13). Importantly, lower end-diastolic volume (EDV), impaired myocardial strain, reduced cardiac output (CO), and hypotension — which occur in the CLP model — can be regulated by natriuretic peptide signaling and are altered in coordination with plasma BNP (10, 13). Although BNP has been shown to regulate blood pressure and cardiac load (10), there is no study that has identified the pathways leading to increased BNP expression in sepsis, and neither has aberrant upregulation of BNP in sepsis been tested as a major therapeutic target for septic hypotension.

Our group has pursued various studies that identified contribution of reduced fatty acid metabolism and impaired mitochondrial function to cardiac dysfunction in sepsis (14–17). We have previously shown that the c-Jun N-terminal kinase (JNK) pathway suppresses gene expression of PPARα, and other proteins related to fatty acid and glucose oxidation, and causes myocardial depression (14). JNK phosphorylates and, hence, activates c-Jun, which is a leucine zipper transcription factor and major constituent of the activating protein–1 (AP-1) complex. Here, we show a potentially novel pathway that associates JNK and c-Jun with pathophysiology of septic hypotension, which constitutes one of the most critical complications of the disease. Specifically, we show that c-Jun, acting downstream of JNK, activates the *Nppb* gene in sepsis and that aberrantly increased plasma BNP contributes to septic hypotension. Furthermore, we found that inhibition of JNK or BNP increased preload and CO in septic mice, increased blood pressure, and improved survival. Taken together, these results identify JNK signaling and BNP as potentially novel therapeutic targets for the treatment of septic hypotension.

## Results

### Genetic ablation of the Nppb gene delays hypotension and increases cardiac preload.

**Figure 1:**
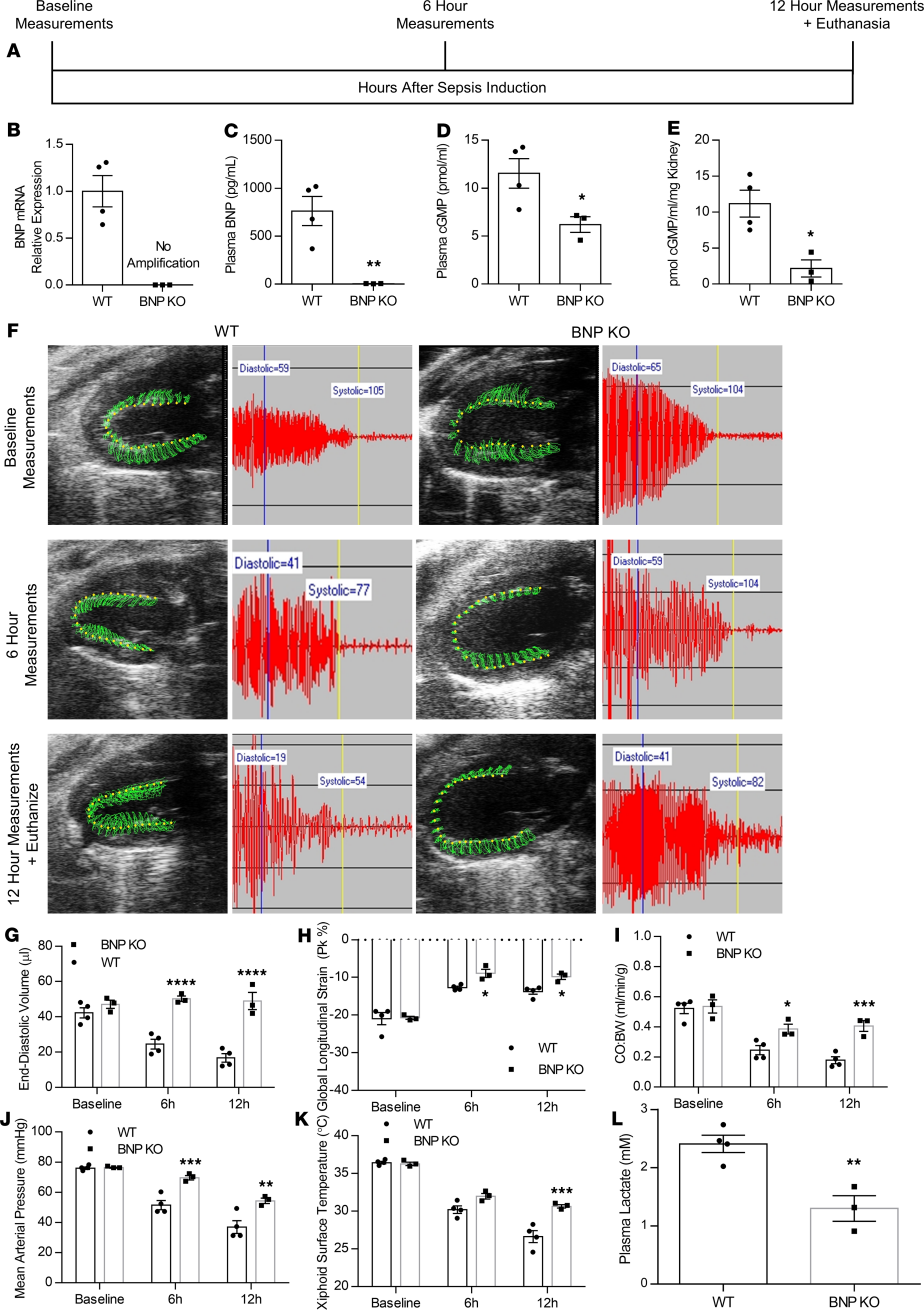
Genetic deletion of the *Nppb* gene delays hypotension in septic mice. (**A**) Experimental outline for CLP surgery in mice with genetic *Nppb* deletion and WT controls. (**B–D**) BNP mRNA (**B**), plasma BNP (**C**), plasma cGMP (**D**), and kidney cGMP (**E**) in BNP-KO mice 12 hours after CLP surgery. *n* = 4 WT, *n* = 3 BNP-KO. *P < 0.05, **P < 0.01 versus WT by *t* test. (**F**) Representative parasternal long-axis view and tail-cuff blood pressure trace in BNP-KO mice and matched controls following CLP. (**G–K**) End-diastolic volume (**G**), global longitudinal strain (**H**), cardiac output normalized to body weight (**I**), mean arterial pressure (**J**), and xiphoidal surface temperature (**K**) at baseline, 6 hours after CLP, and 12 hours after CLP. *P < 0.05, **P < 0.01, ***P < 0.001, ****P < 0.0001 versus WT by 2-way ANOVA for repeated measures with Sidak multiple comparisons. (**L**) Plasma lactate in BNP-KO mice 12 hours after CLP. *n* = 4, WT; *n* = 3, BNP-KO. **P < 0.01 versus WT by *t* test between treatments.

Previous studies have associated BNP with lower blood pressure (18, 19) and have associated increased BNP with tissue hypoxia and mortality in septic patients (9). Furthermore, we previously showed that elevation in BNP following CLP precedes the onset of hypotension and occurs in coordination with reduced CO (13). We therefore investigated potential involvement of BNP in driving hypotension in sepsis. We performed CLP surgery, followed by measurements of cardiac function and blood pressure, in mice with targeted genetic deletion of the *Nppb* gene (BNP-KO; Figure 1A). Deletion of the *Nppb* gene was confirmed by lack of amplification of BNP mRNA by reverse transcription PCR (RT-PCR) in hearts obtained from the BNP-KO mice (Figure 1B) and undetectable plasma BNP levels (Figure 1C). Consistently, we observed a significant reduction in cGMP levels in both plasma (Figure 1D) and the kidneys of (Figure 1E) of mice that underwent CLP surgery. We then performed 2D echo analysis to measure CO normalized to body weight (CO:BW), EDV, and global longitudinal strain (GLS), and we measured blood pressure via tail cuff in BNP-KO mice with CLP (Figure 1F). Interestingly, we observed that, while EDV was reduced in WT controls within 6 hours of CLP surgery, which progressed further by 12 hours, BNP-KO mice did not experience a reduction in EDV, which was significantly increased at 6 and 12 hours compared with WT controls (Figure 1G). Although GLS in BNP-KO mice did not differ significantly compared with WT controls at baseline, we found that GLS was impaired in both septic BNP-KO and septic WT controls at 6 and 12 hours after CLP (Figure 1H). Assessment of CO:BW, which affects blood pressure and is regulated by EDV and GLS (13), showed that BNP-KO mice had significantly higher values (~1.5-fold at 6 and 12 hours) compared with WT control mice at the same time points (Figure 1I). This elevation in CO:BW was associated with significantly increased mean arterial pressure (MAP) at both time points (Figure 1J). Consistently, we found that septic BNP-KO mice had significantly higher body surface temperature (+2°C at 6 hours and +3.5°C at 12 hours after CLP) compared with WT mice that underwent CLP (Figure 1K). The observed improvements in CO:BW, MAP, and body surface temperature of the septic BNP-KO mice were accompanied by significantly reduced plasma lactate levels (Figure 1L), suggesting that genetic BNP ablation protects against polymicrobial sepsis.

### Generation of mouse monoclonal IgG with BNP neutralizing capabilities.

To assess further the therapeutic potential of BNP inhibition in reversing low preload and increasing CO and blood pressure, we developed a neutralizing antibody directed against the mouse BNP-45 peptide. We evaluated the neutralizing capacity of the antibody by assessing in vitro the BNP-mediated production of cGMP in HEK293 cells treated with and without inhibitor of phosphodiesterases (3-isobutyl-1-methylxanthine; IBMX) that metabolize cGMP. Consistently, we found that IBMX increased levels of cGMP in cell lysate, which was further augmented by stimulation with BNP peptide (Supplemental Figure 1A; supplemental material available online with this article; https://doi.org/10.1172/jci.insight.133675DS1). Using this system, hybridoma supernatants were tested for antigen binding via Western blot, as well as for BNP neutralizing capability in HEK293 cells stimulated with 5 μM mouse BNP-45 peptide for 20 minutes. As anticipated, the negative control (NC) supernatant did not affect BNP-induced upregulation of cGMP concentrations (2-fold) (Supplemental Figure 1, B–F). Almost complete suppression of BNP-stimulated cGMP accumulation was observed for 4 supernatants, including 26H10, 27D6, 19B3, and 17G11 (Supplemental Figure 1, B–E). On the other hand, supernatant 18A3 did not suppress cGMP formation (Supplemental Figure 1F). Based on these results, we selected these 5 clones (4 positive and 1 negative) for subcloning and cryopreservation. To select the most efficient antibody for purification and further testing, we performed CLP surgery in C57BL/6 mice and injected i.p. hybridoma supernatant (~10–15 μg for each antibody) at the time of surgery. Since we observed for mice with genetic *Nppb* deletion, injection with BNP neutralizing supernatant delayed hypotension (Supplemental Figure 1G) and increased body surface temperature (Supplemental Figure 1H). Among all antibodies tested, 19B3 had the most profound beneficial effect and, therefore, was selected for purification and application in mouse sepsis models. Following purification, ELISA data provided by the antibody manufacturer revealed that antibody 19B3 had an EC_50_ of 120.2 μg/L (Supplemental Figure 1I).

### BNP neutralization reverses low preload and increases blood pressure in mice with sepsis.

**Figure 2:**
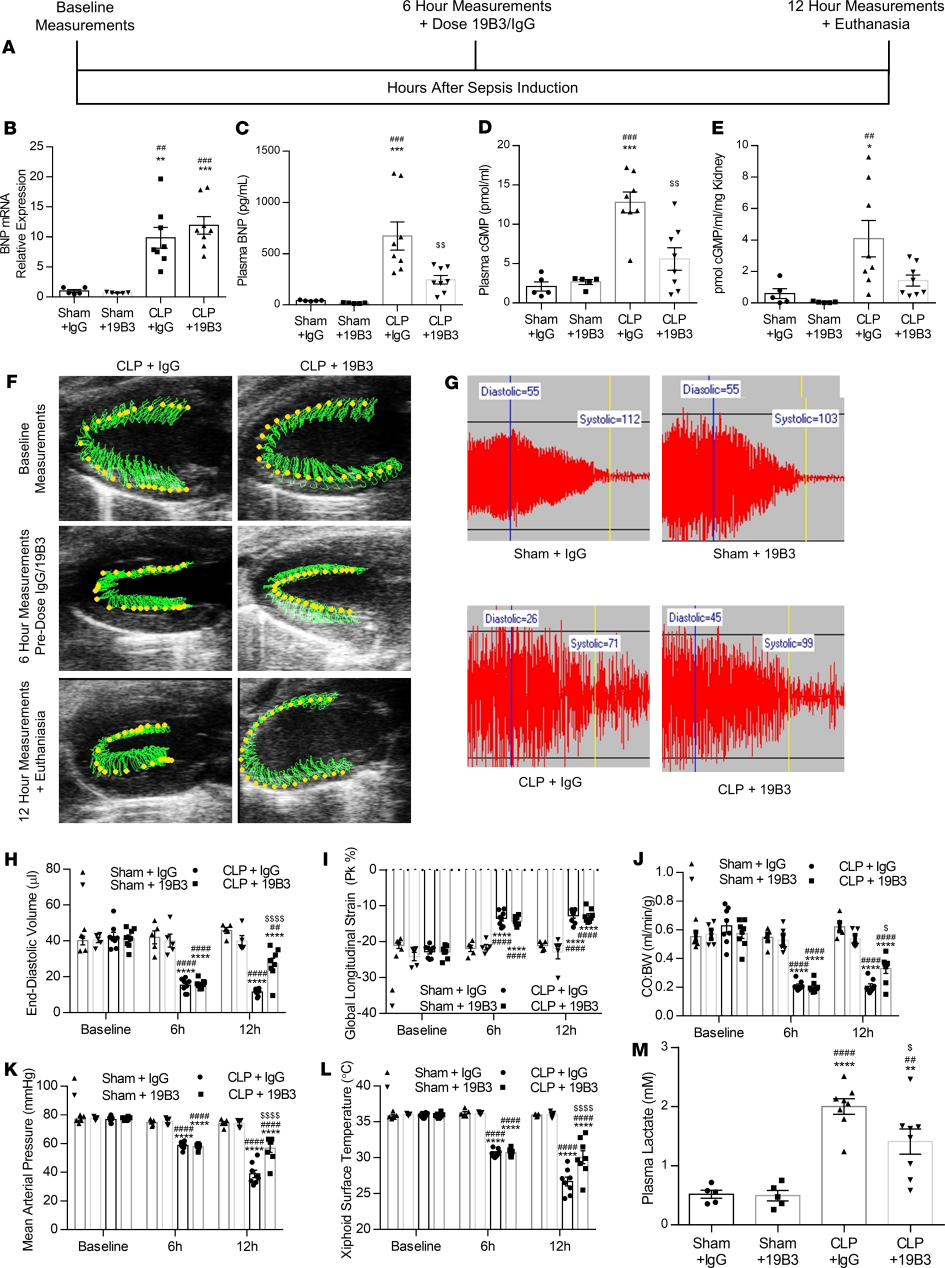
BNP neutralization via 19B3 reverses low preload, increases cardiac output, and protects against hypotension. (**A**) Experimental outline for CLP surgery in mice followed by administration of 19B3 (50 mg/kg) or IgG (50 mg/kg) 6 hours after CLP. (**B–E**) BNP mRNA (**B**), plasma BNP (**C**), plasma cGMP (**D**), and kidney cGMP (**E**) in BNP-KO mice 12 hours after CLP. (**F** and **G**) Representative parasternal long-axis view (**F**) and tail-cuff blood pressure trace (**G**) in mice following sham or CLP surgery, followed by administration of 19B3 (50 mg/kg) or IgG. (**H–M**) End-diastolic volume (**H**), global longitudinal strain (**I**), cardiac output normalized to body weight (**J**), mean arterial pressure (**K**), surface temperature (**L**), and plasma lactate (**M**) in mice 12 hours after CLP and 6 hours after 19B3 administration. *n* = 5, Sham + IgG; *n* = 5, Sham + 19B3; *n* = 8, CLP + IgG; *n* = 8, CLP + 19B3. *P < 0.05, **P < 0.01, ***P < 0.001, ****P < 0.0001 versus Sham + IgG; ^##^*P* < 0.01, ^###^*P* < 0.001, ^####^*P* < 0.0001 versus Sham + 19B3; ^$^*P* < 0.05, ^$$^*P* < 0.01, ^$$$$^*P* < 0.0001 versus CLP + IgG by 2-way ANOVA with Tukey multiple comparisons between treatments.

The previous experiments revealed that BNP neutralization, applied either prior to or at the time of surgery, confers protection and delays the onset of hypotension. Then, we determined if BNP neutralization can reverse established hypotension, as well as if the known role of natriuretic peptides in regulating cardiac load are involved in the pathophysiology of septic hypotension. Thus, we randomized mice to undergo sham or CLP surgery, and we administered them i.p. with 19B3 or control IgG (2 mg/kg) 6 hours CLP. We assessed cardiac function, blood pressure, and temperature at baseline, prior to administering 19B3 or IgG (6 hours after CLP), and 6 hours after administration of the antibody (12 hours after CLP) (Figure 2A). Compared with sham-operated mice, cardiac BNP mRNA expression of septic mice treated with either 19B3 or IgG was increased to the same extent (Figure 2B). Conversely, a trend for reduced detected plasma BNP was observed in animals treated with 19B3 (Figure 2C). Accordingly, treatment of septic mice with 19B3 reduced plasma cGMP (Figure 2D) and kidney cGMP levels (Figure 2E) compared with septic mice that were administered with control IgG.

As administration of 19B3 reduced plasma BNP activity, we sought to determine the effect this has on sepsis pathophysiology. Therefore, we performed echocardiography analysis (Figure 2G) and blood pressure measurements (Figure 2F). CLP reduced significantly EDV in mice treated with IgG. 19B3 increased preload and reversed the reduction in EDV (Figure 2H) but did not have any beneficial effect on GLS of septic mice (Figure 2I). Consistent with the changes in EDV, septic mice that were treated with control IgG had significantly reduced CO:BW compared with sham mice (Figure 2J). On the other hand, septic mice treated with 19B3 had significantly higher CO:BW measurements (Figure 2J), increased MAP at 12 hours (Figure 2K), higher xiphoidal surface temperature at 12 hours (Figure 2L), and lower plasma lactate 12 hours after CLP, compared with mice with sepsis that were treated with control IgG (Figure 2M).

### JNK–c-Jun signaling accounts for increased Nppb expression.

**Figure 3:**
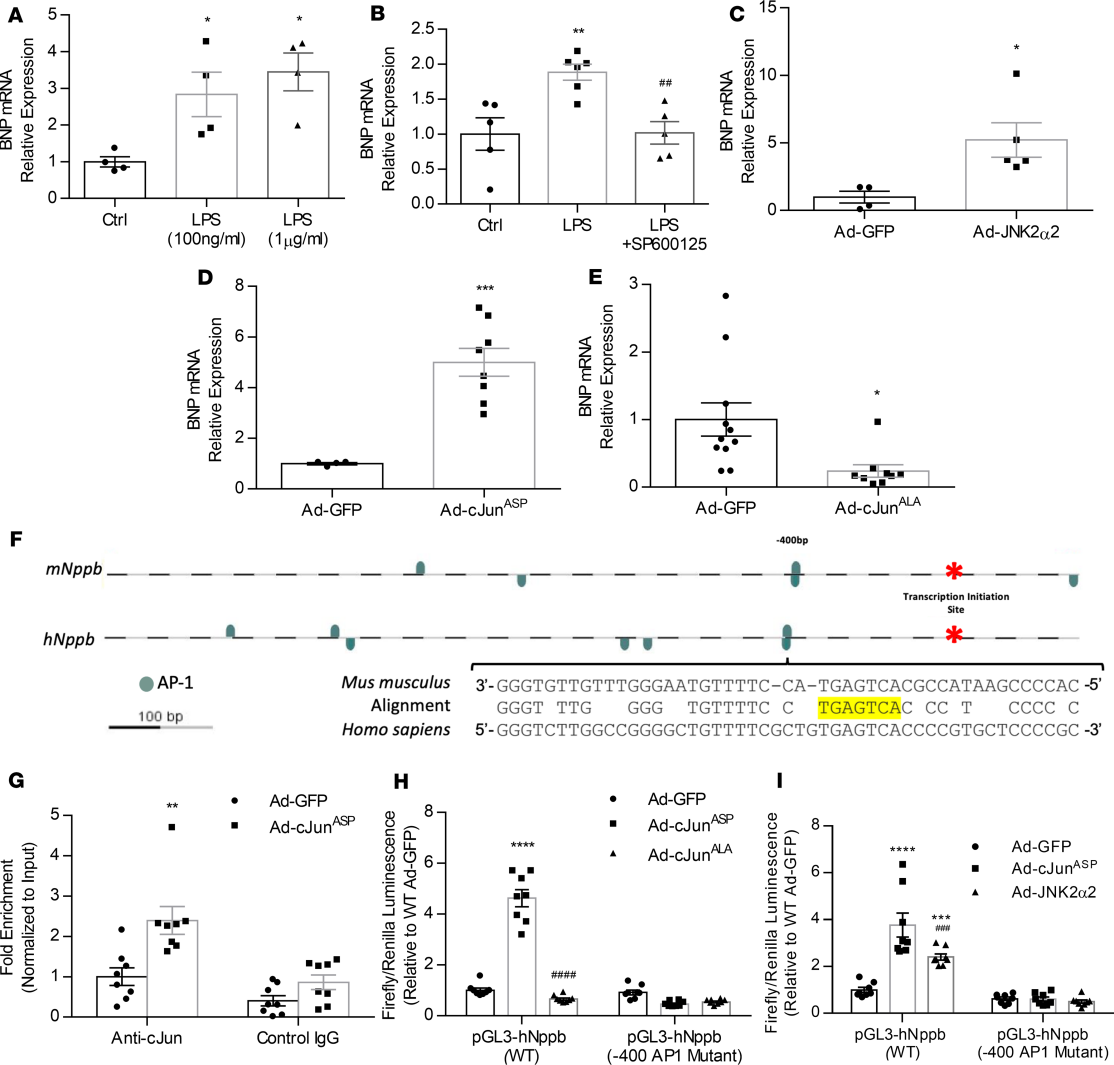
Activation of JNK signaling increases BNP production. (**A**) BNP mRNA levels in AC16 cardiomyocytes stimulated with 100 ng/mL or 1 μg/mL LPS for 8 hours. *n* = 4, control (Ctrl) wells; *n* = 4, LPS (100 ng/mL) wells; *n* = 4, LPS(1 μg/mL) wells. *P < 0.05 versus Ctrl by 1-way ANOVA with Tukey multiple comparisons. (**B**) BNP mRNA levels in AC16 cardiomyocytes stimulated with 1 μg/mL LPS and SP600125 (100nM) for 8 hours. *n* = 5, Ctrl wells; *n* = 6, LPS wells; *n* = 5, LPS + SP600125 wells. **P < 0.01 versus Ctrl, ^##^*P* < 0.01 versus LPS by 1-way ANOVA with Tukey multiple comparisons. (**C**) BNP mRNA levels in AC16 cells infected with Ad-JNK2α2 (MOI, 10; 48 hours). *n* = 4, Ad-GFP wells; *n* = 5, Ad-JNK2α2 wells. *P < 0.05 versus Ad-GFP by *t* test. (**D** and **E**) BNP mRNA levels in AC16 cardiomyocytes infected with Ad–c-Jun^ASP^ (**D**) or Ad–c-Jun^ALA^ (**E**). For **D**, *n* = 4, Ad-GFP wells; *n* = 8, Ad–c-Jun^ASP^ wells. For **E**, *n* = 11, Ad-GFP wells; *n* = 9, Ad–c-Jun^ALA^ wells. *P < 0.05, ***P < 0.001 versus Ad-GFP by *t* test. (**F**) Schematic representation of the mouse *Nppb* and human *NPPB* promoters showing a conserved predicted AP1 motif located –400 bp upstream of the transcription start site, highlighted in yellow. (**G**) Enrichment of the –400 AP1 site of human *NPPB* promoter with c-Jun in chromatin samples from AC16 cells treated with control Ad-GFP or Ad–c-Jun^ASP^ (ChIP-qPCR analysis). *n* = 8 wells/group. **P < 0.01 versus Ad-GFP by *t* test. (**H** and **I**) Firefly luminescence normalized to Renilla luminescence in AC16 cells transfected with pGL3-hNPPB (WT) or –400AP1 mutant, and infected with Ad–c-Jun^ASP^ or Ad–c-Jun^ALA^ (**H**) or with Ad–c-Jun^ASP^ or Ad-JNK2α2 (**I**). *n* = 8 wells/group. ***P < 0.001, ****P < 0.0001 versus Ad-GFP; ^###^*P* < 0.001, ^####^*P* < 0.0001 versus Ad–c-Jun^ASP^ by 1-way ANOVA with Tukey multiple comparisons.

Following confirmation of the involvement of BNP in reducing blood pressure in sepsis, we pursued the signaling pathway that mediates upregulation of *Nppb* expression in sepsis and assessed the therapeutic potential of its inhibition in alleviating BNP upregulation and septic hypotension. In our previous study (14), we had observed inverse correlation of cardiac JNK signaling activation and *Nppb* expression. To assess further the contribution of JNK signaling to increased BNP expression, we treated human AC16 cardiomyocytes with *E*. *coli* LPS, which activates JNK signaling (14). Treatment of AC16 cells with either 100 ng/mL LPS or 1 μg/mL LPS for 6 hours increased BNP mRNA levels compared with vehicle control (Figure 3A). The effect of LPS on BNP expression was abrogated upon combined treatment of AC16 cells with LPS (1 μg/mL) and the pharmacological JNK inhibitor SP600125 (JNKi, 1 μg/mL) (Figure 3B). To confirm that the effect of LPS on BNP expression was dependent on JNK, we infected AC16 cardiomyocytes with adenovirus-encoding constitutively active JNK (Ad-JNK2α2; multiplicity of infection [MOI], 10) (14). Compared with controls infected with Ad-GFP, JNK2α2 BNP mRNA levels increased significantly (5.2-fold) (Figure 3C). Taken together, these results suggest the JNK activation increases BNP transcription.

To elucidate further the mechanism via which JNK activates BNP transcription, we investigated whether c-Jun, which is activated by JNK, is also capable of increasing BNP transcription. Infection of AC16 cardiomyocytes with adenovirus containing c-Jun^ASP^, a constitutively active c-Jun isoform (16) with JNK phosphorylation sites that have been substituted to the phospho-mimetic aspartic acid (Ad–c-Jun^ASP^), increased BNP mRNA levels 5-fold (Figure 3D). On the other hand, treatment of AC16 cells with adenovirus expressing c-Jun^ALA^ that has the same amino acids substituted to alanine, which cannot be phosphorylated (Ad–c-Jun^ALA^), reduced BNP mRNA by 3-fold (Figure 3E).

### c-Jun activates the NPPB promoter via direct binding.

Since we observed that c-Jun activated BNP transcription, we sought to identify the regulatory elements required for c-Jun–mediated BNP stimulation. c-Jun is a leucine zipper transcription factor and a member of the AP-1 complex that binds to the consensus sequence 5′-TGA[G/C]TCA-3′. In silico analysis of the mouse *Nppb* and human *NPPB* promoters (–2000 to +100 bp) for potential AP-1 sites (Genomatix software) identified the presence of 4 on the mouse promoter and 7 on the human promoter (Figure 3F). Alignment of the mouse and human promoters (PromoterWise software) identified a single conserved AP-1 site located –400 bp upstream of the transcription start site of the mouse and human promoter (Figure 3F). ChIP analysis in AC16 cells infected with Ad–c-Jun^ASP^ showed a 3-fold enrichment of the –400 AP-1 site compared with control cells infected with Ad-GFP, thereby confirming that c-Jun binds to the *NPPB* promoter via the –400 AP-1 site (Figure 3G).

To identify if binding of c-Jun to the –400 AP-1 site is important for the activating effect of c-Jun on the *NPPB* promoter, we cloned the WT human *NPPB* promoter upstream from luciferase reporter gene (pGL3-BV vector) (pGL3-*hNPPB* WT) and cotransfected AC16 cells with the pGL3-*hNPPB* WT plasmid and the pTK-Renilla transfection control plasmid. We observed that luminescence was increased 4.6-fold in response to Ad–c-Jun^ASP^ but not Ad–c-Jun^ALA^ (Figure 3H). Mutation of the –400 AP-1 site (pGL3-h*NPPB* Δ-400AP-1; Supplemental Figure 2) ablated the capability of c-Jun^ASP^ to activate the human *NPPB* promoter (Figure 3H). We next sought to determine if the same effect would occur following activation of endogenous c-Jun by infecting AC16 cells with Ad-JNK2α2 (Figure 3I). JNK2α2 overexpression in AC16 cells increased human *NPPB* promoter activity by 2.4-fold, although the increase was to a smaller extent compared with c-Jun^ASP^. As with c-Jun^ASP^, mutation of the AP-1 site in the human *NPPB* promoter negated the ability of JNK2α2 to stimulate luciferase activity (Figure 3I). Taken together, these results suggest that the –400 AP-1 site is critical for mediating activation of the *NPPB* promoter by JNK and c-Jun.

### Pharmacological inhibition of JNK reduces BNP production after CLP.

**Figure 4:**
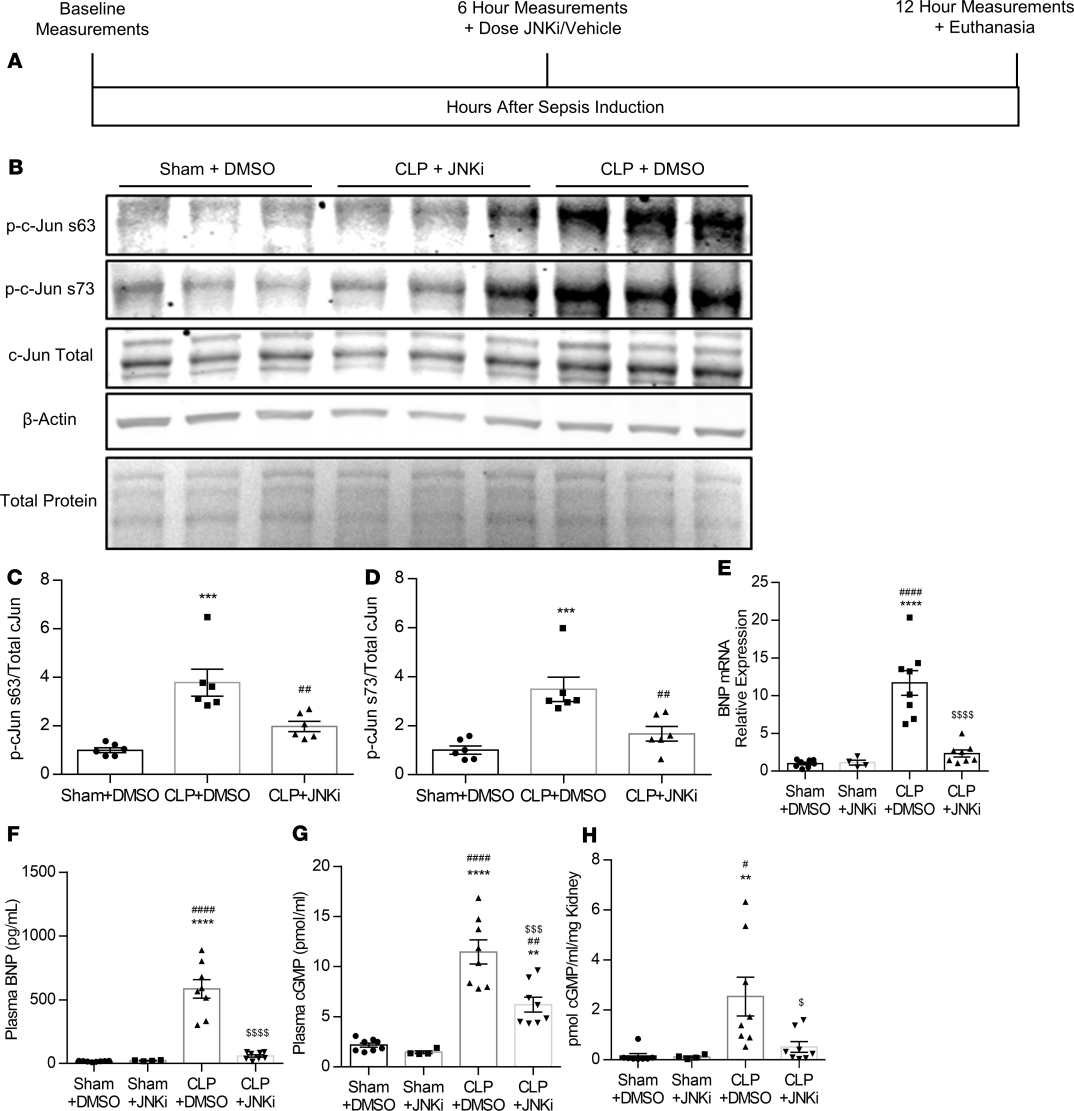
Cardiac JNK signaling regulates BNP in septic mice. (**A**) Experimental design for mice included in the study. (**B–D**) Representative immunoblot (**B**) and densitometric quantification of phospho–c-Jun at Ser63 (**C**) and phospho–c-Jun at Ser73 (**D**). Two separate Western blots with 3 replicates per group were run for a total of *n* = 6 mice/group. ***P < 0.001 versus Sham, ^##^*P* < 0.01 versus CLP + DMSO. (**E–G**) Cardiac BNP mRNA levels (**E**), plasma BNP concentration (**F**), and plasma cGMP levels (**G**) in mice that underwent CLP or sham surgery followed by administration of JNK inhibitor SP600125 (5 mg/kg) or vehicle (DMSO) 6 hours after CLP. (**H**) Kidney cGMP levels normalized to kidney weight in mice that underwent CLP or sham surgery with administration of DMSO or JNKi 6 hours after surgery. *n* = 8, Sham + DMSO; *n* = 4, Sham + JNKi; *n* = 8, CLP + DMSO; *n* = 8, CLP + JNKi. **P < 0.01, ****P < 0.0001 versus Sham + DMSO; ^##^*P* < 0.01, ^####^*P* < 0.0001 versus Sham + JNKi; ^$^*P* < 0.05, ^$$^*P* < 0.01, ^$$$$^*P* < 0.0001 versus CLP + DMSO by one-way ANOVA with Tukey multiple comparisons.

To validate the translational potential of JNK- and c-Jun–mediated upregulation of BNP expression that we observed in AC16 cardiomyocytes, we investigated the effect of pharmacological JNK inhibition on *Nppb* transcription and plasma BNP levels in mice with polymicrobial sepsis. Specifically, we administered the JNKi (5 mg/kg) or control vehicle (DMSO 1 mL/kg) 6 hours after sepsis induction in C57BL/6 mice, when cardiac dysfunction and hypotension have occurred, and followed them until 12 hours after CLP (Figure 4A). JNK inhibition prevented the 4-fold increase in c-Jun phosphorylation at S63 and S73 (Figure 4B) that occurred in untreated septic mice (Figure 4, C and D). Cardiac BNP mRNA was increased 11.9-fold in septic mice compared with control mice with sham surgery (Figure 4E). SP600125 reduced BNP mRNA (5-fold) in mice with CLP (Figure 4E). Consistently, plasma BNP was elevated from 20 pg/mL in sham mice treated with vehicle to 585 pg/mL in vehicle-treated septic mice, and this did not occur in septic mice that were treated with SP600125 (Figure 4F). Suppression of BNP expression by SP600125 was accompanied by impaired BNP signaling, as shown by lower plasma cGMP (Figure 4G) and kidney cGMP levels (Figure 4H), which are normally increased in response to direct administration of BNP (20) and in septic patients (21–23). Thus, BNP is a contributing factor that partially accounts for increased plasma cGMP in sepsis (Figure 4G).

### JNK inhibition increases preload and CO and restores blood pressure in CLP-associated sepsis.

**Figure 5:**
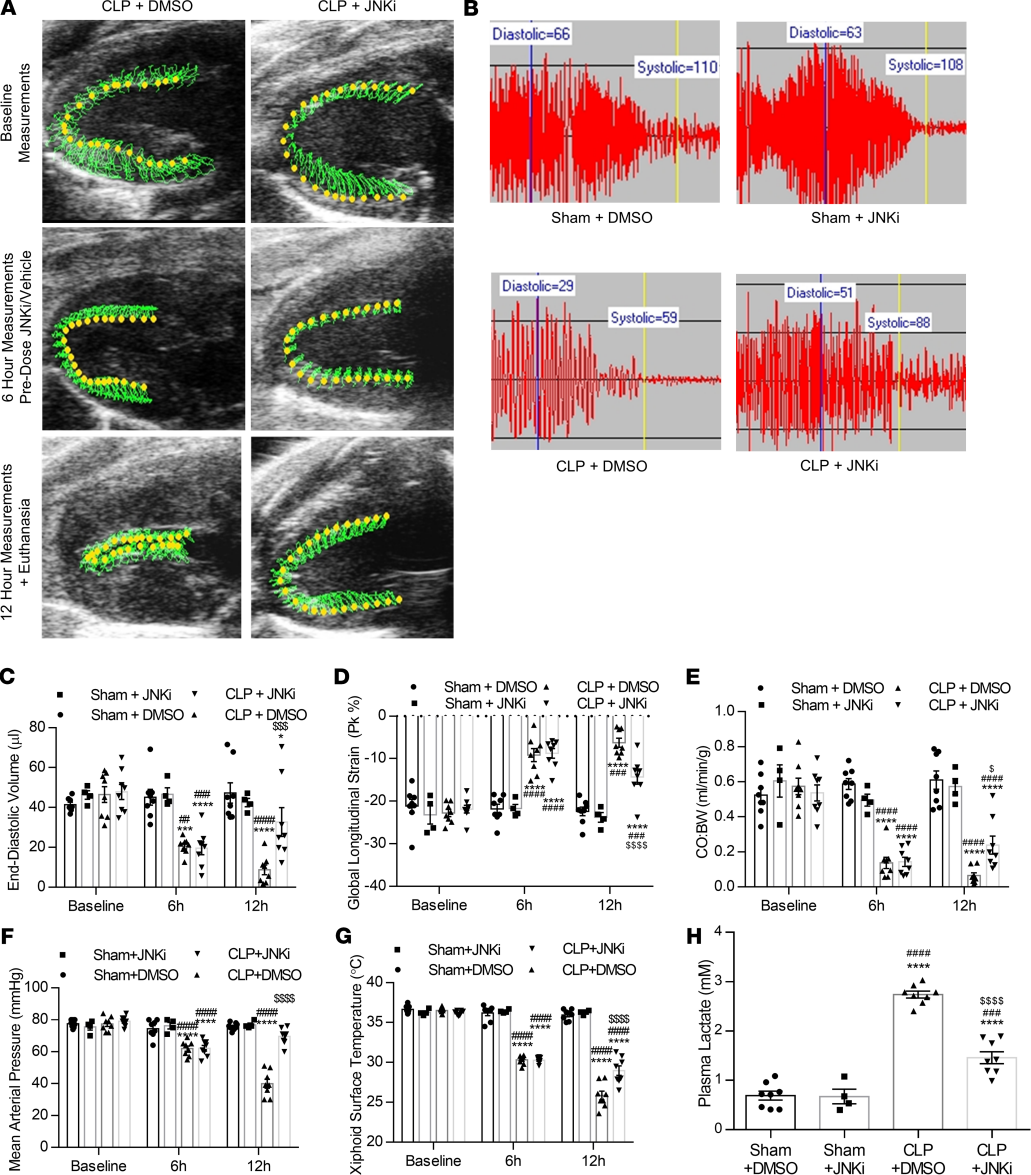
Pharmacological JNK inhibition increases preload and cardiac output and reverses septic hypotension. (**A** and **B**) Representative parasternal long-axis view (**A**) and tail-cuff blood pressure trace (**B**) in mice following sham or CLP surgery followed by administration of SP600125 ( 5mg/kg) or DMSO. (**C–G**) End-diastolic volume (**C**), global longitudinal strain (**D**), cardiac output normalized to body weight (**E**), mean arterial pressure (**F**), and xiphoidal surface temperature (**G**) at baseline, 6 hours after CLP prior to drug/vehicle administration, and 12 hours after CLP and 6 hours after drug/vehicle administration. *n* = 8, Sham + DMSO; *n* = 4, Sham + JNKi; *n* = 8, CLP + DMSO; *n* = 8, CLP + JNKi. *P < 0.05, ***P < 0.001, ****P < 0.0001 versus Sham + DMSO; ^##^*P* < 0.01, ^###^*P* < 0.001, ^####^*P* < 0.0001 versus Sham + JNKi; ^$^*P* < 0.05, ^$$$^*P* < 0.001, ^$$$$^*P* < 0.0001 versus CLP + DMSO by 1-way ANOVA with Tukey multiple comparisons. (**H**) Plasma lactate in mice 12 hours after CLP and 6 hours after drug/vehicle administration at the time of euthanasia. ****P < 0.0001 versus Sham + DMSO; ^###^*P* < 0.001, ^####^*P* < 0.0001 versus Sham + JNKi; ^$$$$^*P* < 0.0001 versus CLP + JNKi by 2-way ANOVA with Tukey multiple comparisons between treatments.

Since we observed that JNK inhibition suppressed after CLP BNP upregulation and decreased plasma cGMP, we investigated whether JNK inhibition can improve blood pressure, preload, and CO, which are known to be affected by BNP signaling. Therefore, we performed speckle-tracking echocardiography to measure cardiac function (Figure 5A) and blood pressure measurement via tail cuff (Figure 5B) in septic mice treated with the JNKi. In accordance with our previous observations (13), mice with CLP experienced a rapid reduction in EDV, which was reduced (2.2-fold) 6 hours after CLP (Figure 5C). EDV increased (1.6-fold) toward baseline following administration of SP600125, whereas vehicle-treated animals experienced further decline in EDV (Figure 5C). We also measured GLS as a marker of cardiac systolic function, which was impaired (2.3-fold) 6 hours after CLP (Figure 5D), as shown previously (13). Treatment with SP600125 increased GLS compared with septic mice that received vehicle (Figure 5D). Since we demonstrated that both reduced EDV and impaired GLS contribute to the decline in CO after CLP, which predicts mortality (13), we measured CO:BW before and after treatment with SP600125. Consistently, we observed that — opposite to septic mice, which had lower CO:BW 6 hours after CLP — CO:BW was increased in septic mice that received SP600125 (Figure 5E).

In accordance with improved CO, treatment with SP600125 increased MAP by 8 mmHg (Figure 5F) and prevented decline in xiphoidal surface temperature compared with vehicle-treated septic mice at 12 hours. However, SP600125 did not reverse hypothermia completely (Figure 5G). Consistently, we observed that SP600125 partially suppressed plasma lactate in animals with CLP that were treated with SP600125 compared with those treated with vehicle (Figure 5H). Thus, pharmacological inhibition of JNK confers a beneficial effect in septic mice by reversing established hypotension and cardiac dysfunction.

### JNK inhibition and BNP neutralization do not reduce kidney injury in septic mice.

Since we observed that both JNK inhibition and BNP neutralization suppressed kidney cGMP levels, we measured plasma blood urea nitrogen (BUN), a clinically relevant marker of kidney dysfunction, to determine if JNK inhibition or BNP inhibition would exert a renal protective effect. Although plasma BUN was elevated in septic mice 12 hours after CLP, none of the 3 experimental setups, BNP-KO mice (Supplemental Figure 3A), and septic C57BL/6 mice treated with either 19B3 (Supplemental Figure 3B) or JNKi (Supplemental Figure 3C) had lower plasma BUN levels compared with their respective septic control mice.

### Both pharmacological JNK inhibition and BNP neutralization increase survival in septic mice.

**Figure 6:**
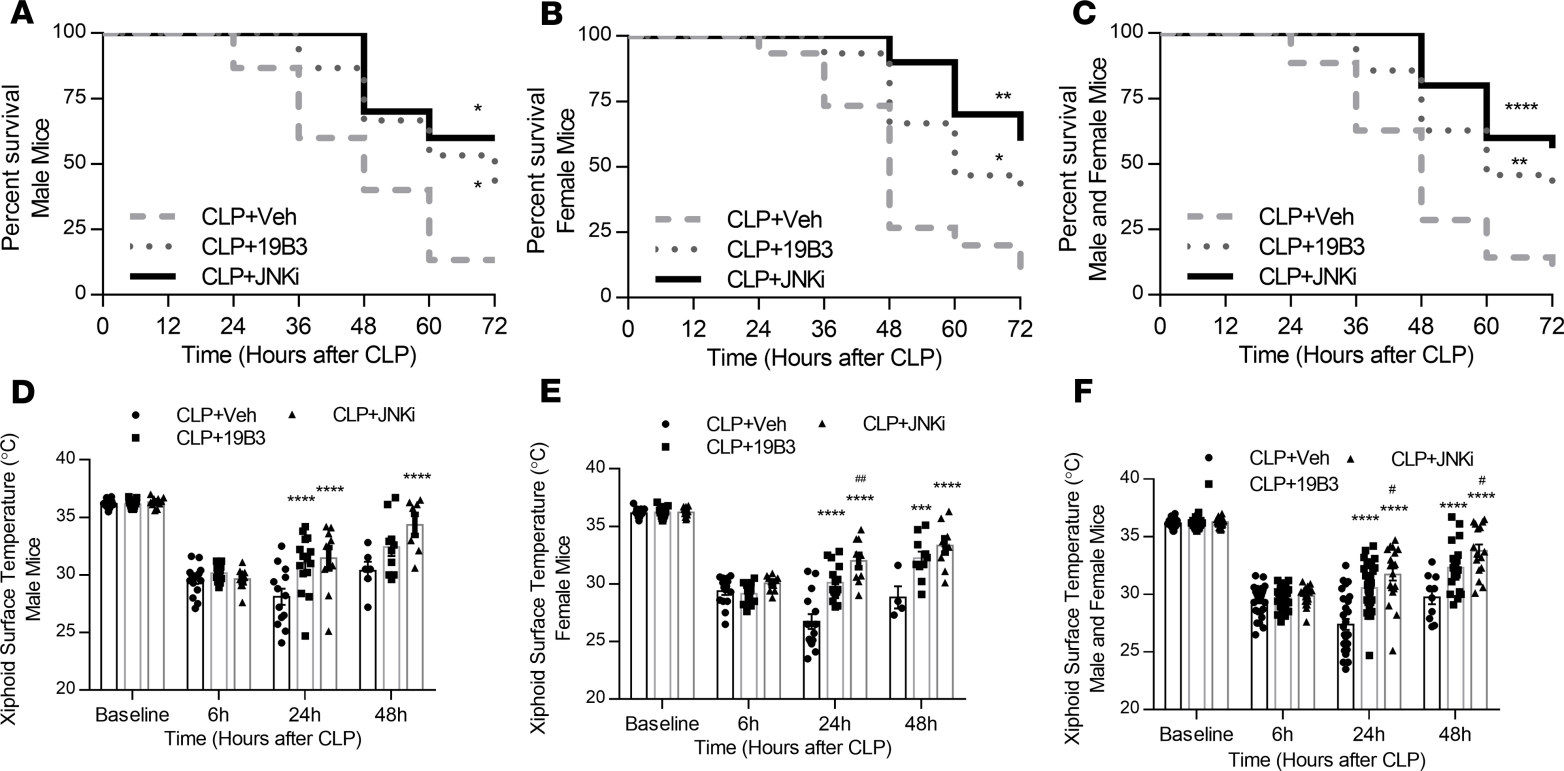
Antibody 19B3 and SP600125 administration increases survival and body temperature in male and female mice treated with antibiotics and fluid. Forty male and 40 female mice were subjected to CLP and assessed for mortality every 12 hours. Mice were allocated beginning 6 hours after CLP to receive a single dose of IgG or 19B3 and a single daily dose of DMSO or SP600125 with saline and ertapenem (70 mg/kg). (**A–C**) Survival plot assessing 72-hour survival in male mice (**A**), female mice (**B**), or mixed-sex (**C**) cohorts. *P < 0.05, **P < 0.01, ****P < 0.0001 versus CLP + Veh. (**D–F**) Body surface temperature in male (**D**), female (**E**), and combined sex (**F**) cohorts of mice followed for survival and 6, 24, and 48 hours after CLP. *n* = 15, CLP + Veh male mice; *n* = 15, CLP + 19B3 male mice; *n* = 10, CLP + JNKi male mice; *n* = 15, CLP + Veh female mice; *n* = 15, CLP + 19B3 female mice; *n* = 10, CLP + JNKi female mice. ***P < 0.001, ****P < 0.0001 versus CLP + Veh; ^#^*P* < 0.05, ^##^*P* < 0.01 versus CLP + 19B3 by 2-way ANOVA with Tukey multiple comparisons between treatments.

We aimed to determine whether JNK inhibition or BNP neutralization, besides acute beneficial effects, provide long-term benefits in survival. Therefore, we assessed survival in male and female mice that were treated with SP600125 or 19B3 and administered daily with ertapenem (70 mg/kg) and saline (20 mL/kg) to mimic treatment of septic patients in an intensive care unit. Mice were subjected to CLP surgery and randomized to receive a single dose of IgG or 19B3 (2 mg/kg) and a daily dose of DMSO or SP600125 (5 mg/kg in 1 mL/kg DMSO) beginning 6 hours after CLP, as follows: IgG + DMSO, 19B3 + DMSO, and IgG + JNKi. Both IgG + JNKi and 19B3 + DMSO groups had significantly reduced mortality compared with IgG + DMSO mice in both male (Figure 6A) and female mice (Figure 6B), as well as in the combined group with both male and female mice (Figure 6C). In accordance with improved survival, xiphoid surface temperature at 6 hours (prior to dosing), 24 hours, and 48 hours after CLP was significantly increased at 24 and 48 hours in male mice (Figure 6D), female mice (Figure 6E), and combined cohorts (Figure 6F) treated with IgG + JNKi or 19B3 + DMSO compared with IgG + DMSO–treated mice. Interestingly, mortality seemed to be lower for female mice compared with male mice within the first 36 hours, but this benefit was lost at 48 hours and on. Also, a significant increase in xiphoidal surface temperature was observed for female mice treated with JNKi compared with 19B3 at 24 hours (Figure 6E), and at 24 and 48 hours when male and female mice were combined (Figure 6F).

## Discussion

Sepsis is a major cause of in-hospital mortality, for which treatment options are limited to supportive therapy and administration of antibiotics (1–3). Hypotension is a major complication in sepsis accounting for increased mortality, and hypotension refractory to treatment is required for the diagnosis of septic shock (3, 6, 7). In the present study, we identified the role of BNP in causing septic hypotension and the involvement of the JNK signaling pathway in the regulation of BNP expression during sepsis. Specifically, we showed that inhibition of either JNK or BNP improved blood pressure in mice with polymicrobial sepsis. Consistently, we observed suppression of plasma lactate production, a clinically relevant measure of sepsis severity and tissue hypoperfusion, which is closely monitored in septic patients (3). The beneficial effects were accompanied by restoration of EDV, increased GLS, and higher CO. Overall, both treatments improved wellness and survival of the septic mice. Taken together, these results demonstrate that pharmacological inhibition of either JNK or BNP exerts multiple beneficial effects in animal models with hypodynamic sepsis.

Our results complement previous animal studies by our group and others, suggesting that JNK inhibition exerts beneficial effects in other aspects of sepsis pathophysiology (14, 24, 25). Specifically, our previous work demonstrated that JNK inhibition in mice with endotoxemia alleviates cardiac dysfunction via stimulation of cardiac fatty acid oxidation (14). Another study, which also used the endotoxemia model, showed that JNK inhibition reduces mortality by alleviating inflammatory signaling pathways (24). One more study assessed the effect of JNK inhibition in the CLP model, which is considered the gold standard of sepsis research. This study demonstrated that JNK inhibition reduces inflammatory injury of the lung and liver, leading to reduced mortality (25). However, neither cardiac function nor blood pressure was assessed in this study. Importantly, our findings indicate a role for JNK inhibition in the stabilization of blood pressure in sepsis, which is mediated, at least in part, by suppression of BNP production.

The involvement of BNP in causality of septic hypotension is a potentially novel finding, which — along with the regulatory role of JNK and c-Jun in *Nppb* expression — holds significant translational potential in sepsis. BNP is a well-described regulator of blood pressure, functioning within a feedback loop to reduce cardiac load in response to cardiac stressors such as ventricular stretch and increased EDV, or hypertension (10–12). Previous studies have shown that transgenic mice overexpressing BNP have reduced resting blood pressure (19, 26). Furthermore, aberrantly increased plasma BNP has been identified as a cause of orthostatic hypotension in patients (18). BNP acts on multiple organ systems to increase venous capacitance, increase natriuresis, and promote plasma shift, the combined effect of which is to reduce the return of blood to the heart. BNP also acts to reduce blood pressure through its effect on arteriolar dilation and suppression of sympathetic outflow (12). Despite the detailed understanding of how BNP acts, the involvement of BNP in septic cardiovascular dysfunction and the therapeutic potential of its inhibition had not been tested. We found that mice with targeted deletion of the *Nppb* gene or interventional administration of a potentially novel BNP neutralizing antibody (19B3) protected against CLP-associated sepsis. These mice had increased EDV and CO, consistent with the volume-reducing effects of BNP, ultimately leading to an increase in MAP. Improvements in EDV in patients with sepsis have been associated with better prognosis (27, 28). Therefore, expansion of EDV in our mice and the resulting increase in CO and blood pressure likely contribute to the improvement in survival we observed following treatment with 19B3.

Compared with WT mice subjected to CLP, BNP-KO mice had reduced GLS, which may be interpreted as worsened systolic dysfunction. This effect was partially mirrored on mice treated with the BNP neutralizing antibody that showed neither worsening nor improvement in GLS. The lack of improvement by BNP inhibition likely results from an increase in blood pressure and expansion of EDV, which would require improvements in cardiac inotropy in order to be reversed. Consistently, measurements of systolic function are known to correlate inversely with blood pressure (29). On the other hand, JNK inhibition may exert a dual effect on the cardiovascular system, resulting from both reduced BNP production and improved cardiac systolic function, which may be accounted for by improved cardiac energetics that we have previously shown in mice with endotoxemia (14, 16). Thus, the beneficial effect of JNK inhibition in GLS is not exclusively attributable to BNP signaling.

We found that activation of the JNK–c-Jun pathway in sepsis stimulates the aberrant increase in BNP despite reduced blood pressure and lower EDV. Other studies that pursued transposase-accessible chromatin analysis (30), sequence analysis (31), and gel shift assays (32) have predicted AP-1 binding sites in the *Nppb* promoter — primarily in response to angiotensin II (AngII) and endothelin-1 (ET1) — that are also involved in blood pressure regulation. AngII signaling, which normally increases arterial pressure, is compromised in sepsis due to lower expression of AngII receptor (33). Thus, BNP upregulation in sepsis seems to be independent from AngII signaling. Surprisingly, higher cardiac BNP expression was associated with increased arterial pressure in healthy rats that received AngII for 6 hours (32), suggesting that the hypertensive effect of AngII can mask the hypotensive effect of BNP. Competition between BNP and AngII may explain the partial improvement (~10 mmHg) that occurred in septic patients treated with AngII (ATHOS-3 trial) (34). An ongoing clinical trial that uses AngII as a vasopressor in patients with sepsis will clarify whether AngII treatment itself and the anticipated partial improvement of arterial pressure will suffice to correct tissue perfusion or whether combined treatment with other interventions that can also increase arterial pressure, such as BNP suppression, is warranted.

Increased plasma ET1, which has also been linked to activation of JNK signaling (35, 36), leads to vasodilation and hypotension and has been associated with morbidity and mortality in sepsis (37). Plasma ET1 levels are higher during endotoxemia (37–40), and infusion of ET1 aggravates kidney dysfunction in pigs with endotoxemia (41). Accordingly, combined blockade of ETA and ETB receptors improves cardiopulmonary function in sheep with endotoxemia (42), improves survival in a mouse model of polymicrobial sepsis (43), maintains renal and cardiac functions in neonatal piglets with endotoxic shock (44), and improves tissue perfusion in pigs with endotoxemia (45, 46). Opposite to selective ETA blockade in septic rats (47), either dual ETA/ETB blockade (47) or single ETB blockade (48) alleviates hypotension in endotoxemic rats, indicating involvement of ETB in signaling that leads to vasodilation in sepsis. However, it remains unknown whether the beneficial effect of ET1 signaling blockade in sepsis involves inhibition of cardiac JNK signaling and eventual BNP suppression.

Previous studies have demonstrated an association between inflammatory cytokine signaling and BNP upregulation. Interestingly, these studies showed that inflammation associated with cardiac allograft rejection results in selective upregulation of BNP but not ANP independent of hemodynamic measurements (49, 50). Stimulation of inflammatory signaling via cytokine administration in cultured cardiomyocytes results in increased BNP expression (51), consistent with our findings in AC16 cardiomyocytes stimulated with LPS. Because JNK signaling is a well-known mediator of inflammatory signaling (25, 52, 53), it is possible that JNK activation may underlie the increase in BNP that has been observed in other inflammatory diseases, such as cardiac rejection. Future studies aimed at understanding the interplay between BNP upregulation and cardiac inflammation using acute and chronic models of inflammation may establish broader implications for our findings in other cardiovascular diseases.

We also found that plasma cGMP was only partially reduced in response to genetic or 19B3-mediated inhibition of BNP. This finding is consistent with other groups that found partial suppression of plasma cGMP and protection against hypotension in endotoxemic mice with genetic deletion of NPR-A (54). These findings suggest that other pathways that signal through cGMP may still be active. Nevertheless, activation of NO signaling and activation of NPR-B by C-type natriuretic peptide, both of which stimulate guanylate cyclase, have previously been identified as major pathophysiological signaling pathways in sepsis (55–57). Sustained activation of these pathways may account for the partial increase in cGMP in BNP-KO mice and in mice treated with SP600125 or 19B3.

The kidneys constitute a major site of action for natriuretic peptide signaling responsible for stimulating natriuresis and reducing blood volume. We found that sepsis increased kidney cGMP levels, and this was abrogated in mice with genetic BNP ablation, as well as mice treated with 19B3 or JNKi. Conversely, plasma BUN levels — markers of kidney function and glomerular filtration, which is elevated following kidney injury in various cases, including sepsis (58) — did not improve by any of our treatments. Thus, although our treatments could act at the level of the kidney to alter natriuresis, the effect of BNP inhibition on CO:BW and MAP seems to precede any potential improvement in kidney function. Because we did not see improvement in kidney function, the effects of JNK inhibition and BNP neutralization in septic mice likely results from reversal of the volume-reducing effects of natriuretic peptides and JNK that act on smooth muscle and endothelial cells to reduce intravascular volume. Importantly, natriuretic peptides and JNK have both been shown to affect vascular function. As a key mediator downstream of inflammatory cytokine signaling, JNK activation has been shown to regulate endothelial activation and increase endothelial permeability, which may partially account for the beneficial effects of JNK inhibition in our septic mice (59–62). Alternatively, natriuretic peptide signaling acts on the vasculature to promote smooth muscle cell relaxation, resulting in arteriolar dilation and increased venous capacitance, and also acts on endothelial cells to increase permeability (12, 63). Future studies are warranted to delineate the detailed effects of BNP inhibition in kidney cGMP levels and acute renal failure in sepsis, as well as to clarify likely interactions with the arterial and venous vasculature, sympathetic nervous system, and the renin-angiotensin-aldosterone system.

Our study included both male and female mice to determine if there were sex-related differences in the response to BNP inhibition or JNK inhibition. Previous studies have suggested that female patients and female mice are protected against sepsis-associated mortality and organ dysfunction (64–66). Conversely, others have found that male and female mice have similar mortality following CLP (67) or that female mice experience higher mortality when sepsis is induced following nephrectomy (68). In our study, we did not observe sex-related differences in end-point mortality or body surface temperature, and both male and female mice responded favorably to SP600125 and 19B3. Female mice showed improved survival only in the early stage of the disease (up to 36 hours). Differences in sepsis induction and postoperative management may account for this discrepancy observed among different studies. Importantly, previous studies that did find a sex-dependent effect used lower severity models and did not apply the same antibiotic treatment as our study, which may have separate sex-specific effects (64, 66). Despite these differences, our study provides strong evidence that activation of JNK and BNP signaling contributes to sepsis-associated mortality, independently of sex-related factors.

Although increased BNP expression in sepsis and the mechanism via that BNP regulates blood pressure have been reported in the past, our study fills a gap in the current state of knowledge on the causality of JNK and BNP in septic hypotension using the current gold-standard mouse model of sepsis. Another study investigated the causative role of NPR in septic hypotension using the endotoxemia model (54). This study found that endotoxemic mice have stable MAP when NPR-A is knocked out; NPR-A serves as the receptor for both BNP and ANP and, therefore, did not attribute pathology to any of the 3 natriuretic peptides, specifically. Furthermore, our study administered JNKi or 19B3 after documenting evidence of cardiovascular dysfunction. Therefore, our study complements the previous one (54) and provide strong evidence that natriuretic peptide signaling may serve as a novel treatment for sepsis.

### Conclusion.

**Figure 7:**
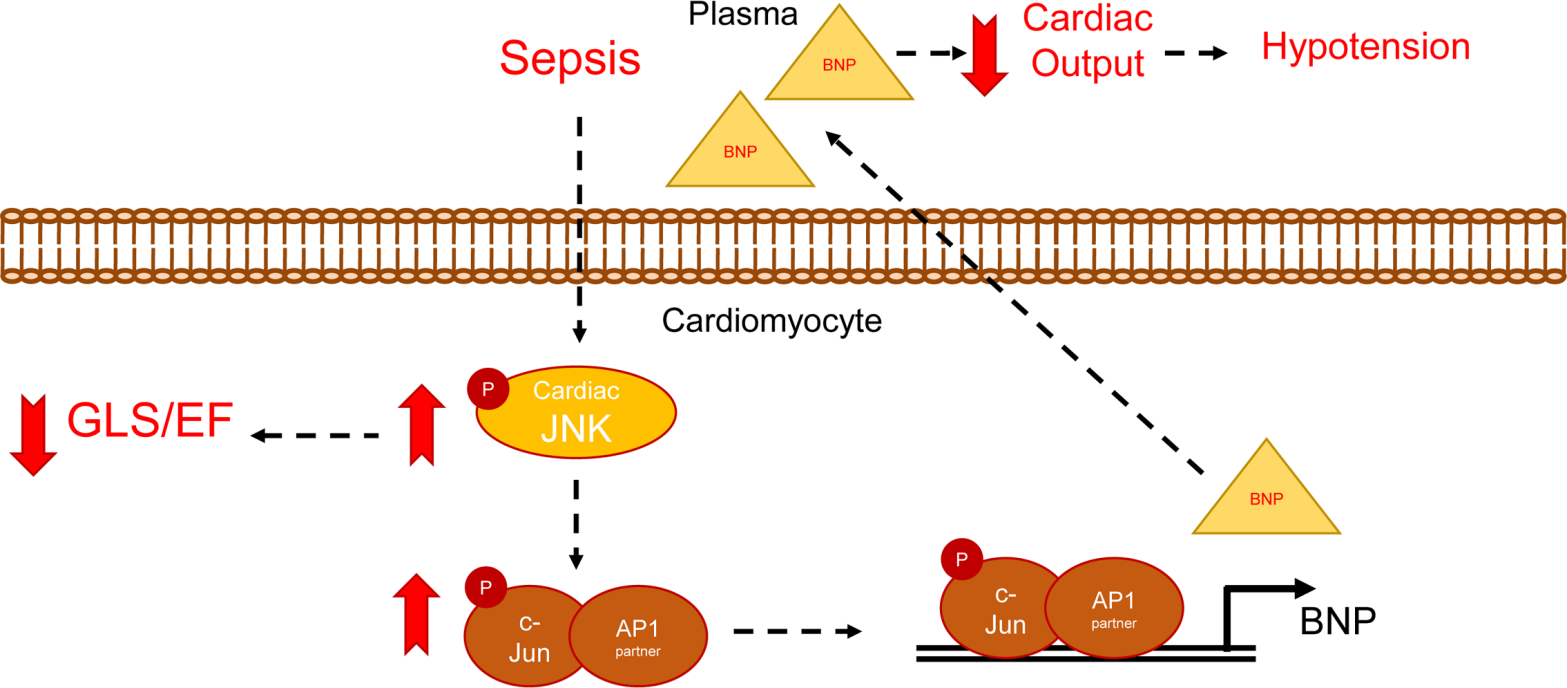
Central illustration. Activation of cardiac JNK in sepsis increases *Nppb* expression and BNP production and causes cardiac systolic dysfunction, both of which contribute to reduced cardiac output and hypotension.

Our findings demonstrate the crucial role that the JNK–c-Jun pathway activation has in the aberrant upregulation of BNP in sepsis and the involvement of the latter in septic hypotension. Accordingly, JNK inhibition and BNP neutralization using clinically relevant dosing strategies improves cardiovascular function, restores blood pressure, and improves 72-hour survival in male and female mice with hypodynamic sepsis. Therefore, the present study provides mechanistic insight into the factors underlying septic shock and proposes the design of 2 treatment strategies to manage critically ill patients with hypodynamic sepsis (Figure 7).

### Limitations.

Echocardiography was performed in rodents using inhaled isoflurane as a sedative. Isoflurane, as with other sedatives, is known to have a cardiorespiratory depressant effect resulting in suppression of heart rate and cardiac contractility (69, 70). To reduce the effects of isoflurane on our measurements, we minimized the level of anesthesia provided to the mice to 0.5% isoflurane and shortened the duration of each imaging session to less than 10 minutes.

In our study, we relied on the use of noninvasive assessment of cardiac function and blood pressure to evaluate the hemodynamic effects of JNK inhibition and BNP neutralization. Although, this approach allows for serial measurements, reducing the effect of catheter insertion on hemodynamics, and eliminates the effect of survival bias, it does not measure ventricular pressures that would help to assess preload. However, we have previously compared our noninvasive approach to invasive assessment of ventricular pressure-volume status and observed that the reduction in volume in our model was associated with reduced preload (13). Importantly, insertion of the catheter resulted in mortality in 3 of 7 of the mice we used in the past for this assessment, therefore introducing a survival bias. Because of this, noninvasive assessment of hemodynamics provides certain benefits for assessing the improvements incurred by JNK inhibition or BNP neutralization in septic mice.

In vitro experiments to investigate the mechanisms underlying BNP upregulation in sepsis were conducted using the AC16 cardiomyocyte cell line. Use of this human cell line offers various benefits including increased stability in cell culture settings, the ability to investigate the role of JNK in regulating BNP in human cells, and the ability to obtain large numbers of cells needed for experiments such as ChIP without interference from noncardiomyocyte cell types that do not express BNP. However, several key differences between AC16 cardiomyocytes and primary cells should be considered when interpreting our results. While our results in AC16 cardiomyocytes are consistent with our in vivo observations, we cannot exclude the possibility that the signaling mechanisms underlying BNP upregulation in AC16 cardiomyocytes are different than those observed in primary cells. Future studies can expand upon our findings to investigate the gene regulatory changes mediated by c-Jun in intact hearts following CLP or in endotoxin-stimulated human induced pluripotent stem cell cardiomyocytes using high-throughput approaches including ChIP sequencing and RNA sequencing.

## Methods

### Animal care and CLP surgery.

WT 7- to 12-week-old male and female C57BL/6 mice weighing between 20–30 g were purchased from the Jackson Laboratory. Global BNP-KO mice were generated as described (71) and maintained on the inbred strain with identical strain-matched WT littermates. Male mice were used for experiments, except for survival challenge, where both male and female mice were used.

CLP was performed as previously described (13, 17, 72). Mice were anesthetized with 3% inhaled isoflurane. Under aseptic conditions, a 1- to 2-cm midline laparotomy was performed, with exposure of the cecum with adjoining intestine. The cecum was tightly ligated below the ileo-cecal valve 1 cm from the distal end and was punctured twice through-and-through with a 19-gauge needle. The length of the ligated cecum was defined as the distance from the distal end of cecum to ligation point, which affects the degree of disease severity. Fecal material was extruded from the punctured cecum, and it was returned to the peritoneal cavity. The peritoneum and the skin were closed with 3 sutures. All mice were resuscitated by injecting s.c. 1 mL of prewarmed 0.9% saline solution, and for postoperative analgesia, the mice received s.c. buprenorphine (0.05 mg/kg body weight, approximately 100 μL fluid in addition) based upon previous studies detailing the CLP procedure (73). Sham mice underwent the procedure to expose the cecum; however, the ligation and puncture steps were omitted.

### Echocardiography.

Cardiac function was assessed by transthoracic echocardiography using the VisualSonics Vevo 2100 system (VisualSonics) at multiple time points, including baseline, 6 hours, and 12 hours after surgery. Prior to echocardiography, hair was removed from each animal’s chest wall using Nair for 1 minute. Sedation was induced with 3% isoflurane and maintained at 0.5% isoflurane during the procedure, which averaged approximately 6–10 minutes per mouse, during which mice were maintained under a lamp on a heated table at approximately 37°C. Parasternal 3-chamber long axis (PLAX) images were taken using the position of the right atrium and posterior mitral valve leaflet as landmarks. Speckle tracking analysis and automatic tracing of PLAX B-mode images was performed by a researcher blinded to the outcome and treatment of the animal. VevoStrain software was used to measure ejection fraction (EF), EDV, end-systolic volume (ESV), stroke volume (SV), and CO using automatic tracing, as well as to detect GLS using speckle tracking echocardiography. For this analysis, B-mode images of 300 frames at greater than 200 frames/second were used. Representative echo images are displayed at baseline, 6 hours, and 12 hours after surgery. Images reflect serial measurements from the same mouse over time. In these images, the green area represents the area covered by the myocardial wall movement between systole and diastole, and the green lines represent the path of the probe during the wall movement. Echo-derived parameters are displayed in Figures 1, 2, and 5, as well as in Supplemental Tables 1–3.

### Noninvasive blood pressure monitoring.

Blood pressure was assessed as previously detailed. Systolic, mean, and diastolic pressure in nonanesthetized animals was measured noninvasively using the BP-2000 tail-cuff system (Visitech Systems). This system uses spectrophotoplethysmographic traces to measure differences in blood flow through the tail during systole and diastole. Prior to randomization, mice were acclimated to the restrainer by taking measurements on 5 different days. Appropriate acclimation to the restrainer is critical to obtaining accurate pressure readings following sepsis induction. During the procedure, animals were placed in the restrainer atop the platform heated to 39°C. Mice were exposed to this environment for at least 10 minutes to allow the tail vasculature to dilate. At least 20 measurements were taken at each time point, and blood pressure curves were assessed to identify appropriate delineation between diastolic and systolic pressures and to ensure reproducibility between measurements. Blood pressure measurements are displayed in Figures 1, 2, and 5, as well as in Supplemental Tables 1–3.

### Infrared thermometer assessment of surface temperature.

Xiphoid surface temperature was assessed using the Etekcity Lasergrip infrared, as previously described (74). Briefly, hair from the xiphoid surface was removed, and mice were restrained. The thermometer was held approximately 3 inches from the xiphoid process, and measurements were recorded at baseline, 6 hours, and 12 hours after surgery.

### Assessment of plasma BNP, L-lactate, and BUN.

Plasma BNP was measured using the Raybiotech BNP ELISA kit (EIAM-BNP) using a 1–4 dilution following the kit instructions. Plasma L-lactate was measured from frozen plasma samples diluted 1:50 with assay buffer using the Sigma Lactate Assay Kit (MAK064) following the kit protocol. Plasma BUN was assayed from plasma diluted 1:20 in distilled water using the Urea Nitrogen (BUN) Colorimetric Detection Kit (Invitrogen EIABUN).

### Measurement of cGMP levels.

Plasma cGMP was measured using the cGMP Enzyme Immunoassy Kit (Sigma-Aldrich, CG201) using plasma diluted 1:20 using the kit instructions. Kidney cGMP was measured in 50 mg of flash frozen kidney tissue that was homogenized in 150 μL of 0.1N HCl (Thermo Fisher Scientific, SA48-1) containing 7.5 mM IBMX (Sigma-Aldrich, 15879). The resulting homogenate was centrifuged at 600 *g* for 10 minutes at 4°C, and the supernatant was applied directly to the cGMP Enzyme Immunoassay Kit (Sigma-Aldrich, CG201). The resulting cGMP concentrations were normalized to kidney tissue weight measured prior to homogenization.

### Cell culture, LPS stimulation, and adenovirus-mediated gene delivery in AC16 cells.

A human ventricular cardiomyocyte–derived cell line, designated AC16 (75), was provided by Mercy Davidson for our in vitro experiments. Cells were maintained in DMEM-nutrient mixture F-12 (DMEM–F-12; Corning, 10-092-CM). AC16 cells were infected with Ad–c-Jun^ASP^, Ad–c-Jun^ALA^, Ad-JNK2α2, and control Ad-GFP. Infections were performed at a MOI of 10 in DMEM-F12 (Thermo Fisher Scientific, 11965118) containing 2% heat-inactivated FBS (HIFBS) for 6 hours. Following infection, cells were washed in sterile PBS and cultured in complete DMEM–F-12. Cells were harvested 48 hours after infection. To mimic activation of inflammatory pathways and endogenous JNK activation in vitro, AC16 cells were stimulated with *E*. *coli* LPS (MilliporeSigma, L2630). Prior to stimulation, cells were cultured in DMEM–F-12 medium without FBS (Peak, PS-FB3) for 2 hours. AC16 cells were stimulated in DMEM–F-12 medium containing 100 ng/mL or 1 μg/mL LPS for 6 hours.

### RNA purification and gene expression analysis.

Total RNA was purified from heart tissue using the TRIzol reagent according to the instructions of the manufacturer (Thermo Fisher Scientific, 15595-026). cDNA synthesis was performed using the Applied Biosystems High Capacity cDNA Reverse Transcription Kit (Applied Biosystems 4368814) from 500 ng of DNase-treated RNA. Quantitative PCR (qPCR) was performed with the SYBR Select Master Mix (Applied Biosystems, 4472903) using the manufacturer recommendations. Incorporation of the SYBR green dye into the PCR products was monitored with the Applied Biosystems StepOnePlus Real-Time PCR System. Samples were normalized against mouse 36B4 or human 18s rRNA. The sequences of the primers used for real-time PCR are shown in Supplemental Table 4.

### Protein purification and Western blot analysis.

Isolated heart tissue was homogenized in radioimmunoprecipitation assay buffer containing protease and phosphatase inhibitors (Pierce Protease and Phosphatase Inhibitor Mini Tablets; Thermo Fisher Scientific, A32959). A total of 30 μg of total protein extract was applied to SDS-PAGE and transferred onto PVDF (Bio-Rad, 170-4275) membranes. Antibodies used for Western blot in this study include rabbit anti–c-Jun (Active Motif, 39309, 1:1000), rabbit anti–phospho–c-Jun at Ser-63 (Cell Signaling Technology, 9261S, 1:1000), rabbit anti–phospho–c-Jun at Ser-73 (Cell Signaling Technology, 9164S, 1:1000), and mouse anti–β-actin (Santa Cruz Biotechnology Inc., sc-47778, 1:200). Pixel density was assessed using ImageJ software (NIH). Density readings were normalized both to β-actin signal intensity and to total protein assessed using Ponceau stain.

### Promoter analysis and ChIP for c-Jun binding to the NPPB promoter.

The human *NPPB* promoter was identified as the region 2000 bp upstream and 100 bp downstream of the transcription start site using genome browser. Predicted AP-1 sites were identified using Genomatix software and PromoterWise software. ChIP experiments were performed as described previously (16), adapted to AC16 cells. In brief, AC16 cells were collected from 15-cm dishes, fixed in 1% paraformaldehyde, and lysed. DNA was sonicated for 20 minutes in total with 30 seconds on/30 seconds off sonication cycles at 80% amplitude in QSonica sonicator. ChIP-grade anti–c-Jun antibody (Active Motif, 39309; 10 μg/sample) was used to precipitate c-Jun–DNA complexes using Protein A/G Magnetic Beads (Pierce). The complexes bound to the beads were washed 6 times with RIPA (RPI, R26200) wash buffer and twice with Tris-EDTA wash buffer (10 mM Tris-HCl, pH8; 1 mM EDTA; 50 mM NaCl), followed by elution (0.1M NaHCO_3_, 1% SDS) and treatment with RNase (MilliporeSigma, R4642) and proteinase K (MilliporeSigma, 71049-3). The cross-links were then reversed, and the DNA was precipitated using ethanol and glycogen for further analysis with qPCR using primers detailed in Supplemental Table 4.

### Generation of pGL3-hNPPB plasmids and site-directed mutagenesis.

The promoter fragment was identified as the 2000 bp upstream and 100 bp downstream of the transcription start site of the *NPPB* gene listed on the genome browser. These sequences were amplified from human DNA isolated from AC16 cells using primers to introduce a 5′ KpnI and 3′ NheI restriction site to the sequence. Primers used to amplify the *NPPB* promoter are detailed in Supplemental Table 2. Successful introduction of the promoter sequence into the pGL3-BV vector was confirmed via diagnostic digestion with KpnI and NheI restriction ezymes (NEB) and sequencing. Site-directed mutagenesis was accomplished using the Q5 Site-Directed Mutagenesis Kit (NEB, E0554S). Primers to mutate the predicted c-Jun binding site to a poly-A sequence were designed using NEBaseChanger tool and are detailed in Supplemental Table 4.

### Luciferase promoter assay.

FuGENE 6 Transfection Reagent (Promega) was used to cotransfect AC16 cells, which were seeded in 96-well plates (10,000 cells/well), with pGL3-*hNppb* (WT or Δ-400AP1, 100ng/well) and pTK-Renilla transfection control (10 ng/well) plasmids according to manufacturer’s protocols. Fourteen hours after transfection, cells were infected with Ad–c-Jun^ASP^, Ad–c-Jun^ALA^, Ad-JNK2α2, or Ad-GFP (MOI, 10) for 6 hours in DMEM–F-12 medium containing 2% HIFBS. Two days after infection, luciferase activity was quantified in cell lysates (Dual-Luciferase Reporter Assay System; Promega, E1910) using the Infinite M1000 PRO plate reader. Graphs are shown depicting firefly luminescence driven by the *NPPB* promoter normalized to Renilla control luminescence.

### Measurement of BNP-stimulated cGMP accumulation in HEK293 cells.

For BNP-stimulated cGMP accumulation, 10,000 HEK293 cells/well of a 96-well plate were cultured in DMEM supplemented with 10% FBS and 1% penicillin-streptomycin (Corning, 30-002-Cl). Prior to BNP stimulation, cells were cultured in FBS-free DMEM for 2 hours, followed by wash with 250 μL Krebs-Ringer Bicarbonate Buffer with glucose (KRBG), pH 7.4, and treatment with 100 μL of freshly prepared stimulation buffer (7.5 mM IBMX in KRBG; MilliporeSigma, 15879) or KRBG vehicle for 10 minutes. Following incubation, cells were stimulated with mouse BNP-45 (Bachem, 4095506) dissolved in 50 μL KRBG at the indicated concentrations for 20 minutes. Cells were lysed by adding 50 μL of 0.4N HCl, and the resulting lysate was acetylated and applied to the cGMP indirect ELISA (MilliporeSigma, CG201) as described in the kit protocol.

### Development of BNP neutralizing mAb.

We used the GenScript custom mAb development service for generating an antibody against the epitope CFGHKIDRIGSVSRLG that is directed against regions of the BNP peptide loop structure required for binding to the NPR. Hybridoma supernatants were screened for their ability to bind to mouse BNP-45 by Western blot, suppress BNP-45–stimulated cGMP accumulation in cell culture, and increase blood pressure and survival in mice after CLP. For Western blotting, 20 ng of purified BNP-45 was separated on SDS-PAGE and transferred to PVDF. Hybridoma supernatant was incubated at a dilution of 1:10 in TBS (Bioworld, 42020056). For BNP-stimulated cGMP accumulation, BNP-45 was rotated at room temperature with hybridoma supernatant diluted 1:2. The BNP-supernatant mixture was used to stimulate HEK293 cells and measure cGMP production by indirect ELISA (MilliporeSigma, cG201). For in vivo treatment with hybridoma supernatant, C57BL/6 male mice were treated i.p. with 1 mL of hybridoma supernatant 1 hour prior to CLP surgery (approximately 10–15 μg of antibody). Blood pressure measurements were taken 6 and 12 hours after surgery. Through this analysis, hybridoma 19B3 (Genscript Custom mAb Service) was selected as the most successful inhibitor of BNP activity and was purified by GenScript, which also assessed affinity of 19B3 to BNP.

### Administration of SP600125 or mAb 19B3 and blinding procedure.

SP600125 (Calbiochem, 420119) was dissolved in DMSO to a concentration of 5 mg/mL. Dissolved SP600125 or vehicle DMSO was administered i.p. at a volume of 1 mL/kg to provide a dose of 5 mg/kg SP600125. 19B3 or mouse IgG (GenScript, A01007) was dissolved in saline to a concentration of 1 mg/mL. Dissolved antibody was administered i.p. at a volume of 2 mL/kg to provide a dose of 2 mg/kg 19B3 or IgG. To ensure appropriate blinding and reduce bias, interventions were administrated by an investigator without knowledge of the identity of the intervention or vehicle provided, and subsequent analyses were performed prior to revealing the identity of the intervention.

### Assessment of survival.

To assess the impact of JNK inhibition or 19B3 on survival, 40 male and 40 female mice were randomized to groups receiving vehicle, 19B3, and JNKi. To allow comparison between all 3 groups, mice were treated with a single dose of either IgG or 19B3 (2 mg/kg) at 6 hours and a single daily dose of SP600125 (5 mg/kg) or DMSO (1 mL/kg) beginning 6 hours after CLP. Control mice received both IgG and DMSO. Mice treated with the antibody received 19B3 and DMSO, while mice treated with the JNKi received SP600125 and IgG. To mimic the treatment conditions of Intensive Care Unit patients as closely as possible, all mice were resuscitated with 20 mL/kg prewarmed saline and were treated with the antibiotic ertapenem (Merck, SML1238; 70 mg/kg) at 6, 24, and 48 hours after CLP. In addition to following survival, body surface temperature measurements were acquired at 6, 24, and 48 hours after CLP prior to administering antibiotics and saline for the day.

### Statistics.

Graphs were created and statistical comparisons were assessed using GraphPad Prism6 software. Data represents mean ± SEM. Analyses were performed using 2-sided Student’s *t* test, 2-way ANOVA with Tukey multiple comparisons to compare groups within each time point, 2-way ANOVA for repeated measures with Sidak multiple comparisons, or 1-way ANOVA with Tukey multiple comparisons. Survival analysis was analyzed using pairwise log-rank (Mantel-Cox) test. Specific statistical tests used are stated within the figure legends. A *P* value less than 0.05 was considered significant.

### Study approval.

Animal protocols were approved by the Temple University IACUC and were carried out in accordance with the *Guide for the Care and Use of Laboratory Animals* (National Academies Press, 2011).

## Author contributions

The research plan was conceived by MH and KD. The methods were performed by MH, IDK, and AD. The analysis of the results was performed by MH, IDK, AD, and KD. *Nppb^–/–^* mice were generated by SKM. Funding was obtained by KD, WJK, MH, and IDK. The original draft was written by MH and KD, while IDK, AD, SKM, and WJK reviewed and edited it. Research supervision was conducted by KD.

## Supplementary Material

Supplemental data

## Version 1 04/23/2020

Electronic publication

## References

[1] Gotts JE, Matthay MA (2016). Sepsis: pathophysiology and clinical management. BMJ.

[2] Marshall JC (2014). Why have clinical trials in sepsis failed?. Trends Mol Med.

[3] Singer M (2016). The Third International Consensus Definitions for Sepsis and Septic Shock (Sepsis-3). JAMA.

[4] Perman SM (2011). Relationship between B-type natriuretic peptide and adverse outcome in patients with clinical evidence of sepsis presenting to the emergency department. Acad Emerg Med.

[5] Post F, Weilemann LS, Messow CM, Sinning C, Münzel T (2008). B-type natriuretic peptide as a marker for sepsis-induced myocardial depression in intensive care patients. Crit Care Med.

[6] Maheshwari K (2018). The relationship between ICU hypotension and in-hospital mortality and morbidity in septic patients. Intensive Care Med.

[7] Gross PA (2006). Hypotension and mortality in septic shock: the “golden hour”. Crit Care Med.

[8] Bai YL, Hu BL, Wen HC, Zhang YL, Zhu JJ (2018). Prognostic value of plasma brain natriuretic peptide value for patientswith sepsis: A meta-analysis. J Crit Care.

[9] Rivers EP, McCord J, Otero R, Jacobsen G, Loomba M (2007). Clinical utility of B-type natriuretic peptide in early severe sepsis and septic shock. J Intensive Care Med.

[10] Kerkelä R, Ulvila J, Magga J (2015). Natriuretic Peptides in the Regulation of Cardiovascular Physiology and Metabolic Events. J Am Heart Assoc.

[11] Ichiki T, Huntley BK, Sangaralingham SJ, Burnett JC (2015). Pro-Atrial Natriuretic Peptide: A Novel Guanylyl Cyclase-A Receptor Activator That Goes Beyond Atrial and B-Type Natriuretic Peptides. JACC Heart Fail.

[12] Potter LR, Abbey-Hosch S, Dickey DM (2006). Natriuretic peptides, their receptors, and cyclic guanosine monophosphate-dependent signaling functions. Endocr Rev.

[13] Hoffman M (2019). Myocardial Strain and Cardiac Output are Preferable Measurements for Cardiac Dysfunction and Can Predict Mortality in Septic Mice. J Am Heart Assoc.

[14] Drosatos K (2011). Inhibition of c-Jun-N-terminal kinase increases cardiac peroxisome proliferator-activated receptor alpha expression and fatty acid oxidation and prevents lipopolysaccharide-induced heart dysfunction. J Biol Chem.

[15] Drosatos K (2013). Peroxisome proliferator-activated receptor-γ activation prevents sepsis-related cardiac dysfunction and mortality in mice. Circ Heart Fail.

[16] Drosatos K (2016). Cardiac Myocyte KLF5 Regulates Ppara Expression and Cardiac Function. Circ Res.

[17] Joseph LC (2017). Inhibition of NADPH oxidase 2 (NOX2) prevents sepsis-induced cardiomyopathy by improving calcium handling and mitochondrial function. JCI Insight.

[18] Krishnan B, Patarroyo-Aponte M, Duprez D, Pritzker M, Missov E, Benditt DG (2015). Orthostatic hypotension of unknown cause: Unanticipated association with elevated circulating N-terminal brain natriuretic peptide (NT-proBNP). Heart Rhythm.

[19] Nakagawa M (2001). Monoclonal antibody against brain natriuretic peptide and characterization of brain natriuretic peptide-transgenic mice. J Hypertens.

[20] Oyama MA, Solter PF, Thorn CL, Stern JA (2017). Feasibility, safety, and tolerance of subcutaneous synthetic canine B-type natriuretic peptide (syncBNP) in healthy dogs and dogs with stage B1 mitral valve disease. J Vet Cardiol.

[21] García-Unzueta MT (1998). High intraplatelet cGMP levels in human sepsis. Clin Microbiol Infect.

[22] Hartemink KJ, Groeneveld AB, de Groot MC, Strack van Schijndel RJ, van Kamp G, Thijs LG (2001). alpha-atrial natriuretic peptide, cyclic guanosine monophosphate, and endothelin in plasma as markers of myocardial depression in human septic shock. Crit Care Med.

[23] Schneider F, Lutun P, Couchot A, Bilbault P, Tempé JD (1993). Plasma cyclic guanosine 3′-5′ monophosphate concentrations and low vascular resistance in human septic shock. Intensive Care Med.

[24] Prasad KD, Trinath J, Biswas A, Sekar K, Balaji KN, Guru Row TN (2014). Anthrapyrazolone analogues intercept inflammatory JNK signals to moderate endotoxin induced septic shock. Sci Rep.

[25] Pizzino G (2015). Blockade of the JNK signalling as a rational therapeutic approach to modulate the early and late steps of the inflammatory cascade in polymicrobial sepsis. Mediators Inflamm.

[26] Ogawa Y (1994). Molecular cloning of the complementary DNA and gene that encode mouse brain natriuretic peptide and generation of transgenic mice that overexpress the brain natriuretic peptide gene. J Clin Invest.

[27] Zanotti Cavazzoni SL, Guglielmi M, Parrillo JE, Walker T, Dellinger RP, Hollenberg SM (2010). Ventricular dilation is associated with improved cardiovascular performance and survival in sepsis. Chest.

[28] Furian T (2012). Ventricular dysfunction and dilation in severe sepsis and septic shock: relation to endothelial function and mortality. J Crit Care.

[29] Boissier F (2017). Left ventricular systolic dysfunction during septic shock: the role of loading conditions. Intensive Care Med.

[30] van Duijvenboden K (2019). Conserved NPPB+ Border Zone Switches From MEF2- to AP-1-Driven Gene Program. Circulation.

[31] Grépin C, Dagnino L, Robitaille L, Haberstroh L, Antakly T, Nemer M (1994). A hormone-encoding gene identifies a pathway for cardiac but not skeletal muscle gene transcription. Mol Cell Biol.

[32] Majalahti T (2007). Cardiac BNP gene activation by angiotensin II in vivo. Mol Cell Endocrinol.

[33] Corrêa TD, Takala J, Jakob SM (2015). Angiotensin II in septic shock. Crit Care.

[34] Khanna A (2017). Angiotensin II for the Treatment of Vasodilatory Shock. N Engl J Med.

[35] Gadea A, Schinelli S, Gallo V (2008). Endothelin-1 regulates astrocyte proliferation and reactive gliosis via a JNK/c-Jun signaling pathway. J Neurosci.

[36] Wang X, Tokuda H, Hirade K, Kozawa O (2002). Stress-activated protein kinase/c-Jun N-terminal kinase (JNK) plays a part in endothelin-1-induced vascular endothelial growth factor synthesis in osteoblasts. J Cell Biochem.

[37] Pan C (2012). Low tidal volume protects pulmonary vasomotor function from “second-hit” injury in acute lung injury rats. Respir Res.

[38] Kaszaki J, Wolfárd A, Boros M, Baranyi L, Okada H, Nagy S (1997). Effects of antiendothelin treatment on the early hemodynamic changes in hyperdynamic endotoxemia. Acta Chir Hung.

[39] Piechota-Polańczyk A, Gorąca A (2012). Influence of specific endothelin-1 receptor blockers on hemodynamic parameters and antioxidant status of plasma in LPS-induced endotoxemia. Pharmacol Rep.

[40] Piechota-Polanczyk A, Kleniewska P, Gorąca A (2012). The influence of ETA and ETB receptor blockers on LPS-induced oxidative stress and NF-κB signaling pathway in heart. Gen Physiol Biophys.

[41] Fenhammar J (2011). Endothelin receptor A antagonism attenuates renal medullary blood flow impairment in endotoxemic pigs. PLoS One.

[42] Kuklin V (2005). Tezosentan-induced attenuation of lung injury in endotoxemic sheep is associated with reduced activation of protein kinase C. Crit Care.

[43] Iskit AB, Senel I, Sokmensuer C, Guc MO (2004). Endothelin receptor antagonist bosentan improves survival in a murine caecal ligation and puncture model of septic shock. Eur J Pharmacol.

[44] Chin A, Radhakrishnan J, Fornell L, John E (2002). Effects of tezosentan, a dual endothelin receptor antagonist, on the cardiovascular and renal systems of neonatal piglets during endotoxic shock. J Pediatr Surg.

[45] Andersson A, Fenhammar J, Frithiof R, Weitzberg E, Sollevi A, Hjelmqvist H (2008). Mixed endothelin receptor antagonism with tezosentan improves intestinal microcirculation in endotoxemic shock. J Surg Res.

[46] Fenhammar J (2008). The endothelin receptor antagonist tezosentan improves renal microcirculation in a porcine model of endotoxemic shock. Acta Anaesthesiol Scand.

[47] Hirata Y, Ishimaru S (2002). Effects of endothelin receptor antagonists on endothelin-1 and inducible nitric oxide synthase genes in a rat endotoxic shock model. Clin Sci.

[48] Nitescu N, Grimberg E, Ricksten SE, Herlitz H, Guron G (2008). Endothelin B receptors preserve renal blood flow in a normotensive model of endotoxin-induced acute kidney dysfunction. Shock.

[49] Wu AH, Johnson ML, Aaronson KD, Gordon D, Dyke DB, Koelling TM (2005). Brain natriuretic peptide predicts serious cardiac allograft rejection independent of hemodynamic measurements. J Heart Lung Transplant.

[50] Ogawa T, de Bold AJ (2012). Brain natriuretic Peptide production and secretion in inflammation. J Transplant.

[51] Ma KK, Ogawa T, de Bold AJ (2004). Selective upregulation of cardiac brain natriuretic peptide at the transcriptional and translational levels by pro-inflammatory cytokines and by conditioned medium derived from mixed lymphocyte reactions via p38 MAP kinase. J Mol Cell Cardiol.

[52] Roy PK, Rashid F, Bragg J, Ibdah JA (2008). Role of the JNK signal transduction pathway in inflammatory bowel disease. World J Gastroenterol.

[53] Verrecchia F, Tacheau C, Wagner EF, Mauviel A (2003). A central role for the JNK pathway in mediating the antagonistic activity of pro-inflammatory cytokines against transforming growth factor-beta-driven SMAD3/4-specific gene expression. J Biol Chem.

[54] Panayiotou CM, Baliga R, Stidwill R, Taylor V, Singer M, Hobbs AJ (2010). Resistance to endotoxic shock in mice lacking natriuretic peptide receptor-A. Br J Pharmacol.

[55] Titheradge MA (1999). Nitric oxide in septic shock. Biochim Biophys Acta.

[56] Koch A (2011). Prognostic value of circulating amino-terminal pro-C-type natriuretic peptide in critically ill patients. Crit Care.

[57] Pernomian L (2016). C-Type Natriuretic Peptide Induces Anti-contractile Effect Dependent on Nitric Oxide, Oxidative Stress, and NPR-B Activation in Sepsis. Front Physiol.

[58] Zarjou A, Agarwal A (2011). Sepsis and acute kidney injury. J Am Soc Nephrol.

[59] Modur V, Zimmerman GA, Prescott SM, McIntyre TM (1996). Endothelial cell inflammatory responses to tumor necrosis factor alpha. Ceramide-dependent and -independent mitogen-activated protein kinase cascades. J Biol Chem.

[60] Pang J, Hu P, Wang J, Jiang J, Lai J (2019). Vorapaxar stabilizes permeability of the endothelial barrier under cholesterol stimulation via the AKT/JNK and NF‑κB signaling pathways. Mol Med Rep.

[61] Xu C, Wu X, Hack BK, Bao L, Cunningham PN (2015). TNF causes changes in glomerular endothelial permeability and morphology through a Rho and myosin light chain kinase-dependent mechanism. Physiol Rep.

[62] Wadgaonkar R (2004). Regulation of c-Jun N-terminal kinase and p38 kinase pathways in endothelial cells. Am J Respir Cell Mol Biol.

[63] Kerkelä R, Ulvila J, Magga J (2015). Natriuretic Peptides in the Regulation of Cardiovascular Physiology and Metabolic Events. J Am Heart Assoc.

[64] Chen J, Chiazza F, Collino M, Patel NS, Coldewey SM, Thiemermann C (2014). Gender dimorphism of the cardiac dysfunction in murine sepsis: signalling mechanisms and age-dependency. PLoS One.

[65] Angele MK, Pratschke S, Hubbard WJ, Chaudry IH (2014). Gender differences in sepsis: cardiovascular and immunological aspects. Virulence.

[66] Wehrenpfennig P, Drechsler S, Weixelbaumer KM, Bahrami S, Osuchowski MF (2014). Mouse model of posttraumatic abdominal sepsis: survival advantage of females over males does not depend on the cecum size. Eur Surg Res.

[67] Kennedy LH, Hwang H, Wolfe AM, Hauptman J, Nemzek-Hamlin JA (2014). Effects of buprenorphine and estrous cycle in a murine model of cecal ligation and puncture. Comp Med.

[68] MacMillan-Crow LA, Mayeux PR (2018). Female mice exhibit less renal mitochondrial injury but greater mortality using a comorbid model of experimental sepsis. Intern Med Rev (Wash D C).

[69] Yange CF (2013). Dose-dependent effects of isoflurane on cardiovascular function inrats. Tzu Chi Medical Journal.

[70] Constantinides C, Mean R, Janssen BJ (2011). Effects of isoflurane anesthesia on the cardiovascular function of the C57BL/6 mouse. ILAR J.

[71] Mishra SK, Hoon MA (2013). The cells and circuitry for itch responses in mice. Science.

[72] Kokkinaki D (2019). Chemically synthesized Secoisolariciresinol diglucoside (LGM2605) improves mitochondrial function in cardiac myocytes and alleviates septic cardiomyopathy. J Mol Cell Cardiol.

[73] Toscano MG, Ganea D, Gamero AM (2011). Cecal ligation puncture procedure. J Vis Exp.

[74] Laitano O (2018). Xiphoid Surface Temperature Predicts Mortality in a Murine Model of Septic Shock. Shock.

[75] Davidson MM (2005). Novel cell lines derived from adult human ventricular cardiomyocytes. J Mol Cell Cardiol.

