# Supplementary material for: B-type natriuretic peptide is upregulated by c-Jun N-terminal kinase and contributes to septic hypotension
Source: JCI Insight. 2020 Apr 23;5(8):e133675. doi: 10.1172/jci.insight.133675 (PMC7205432; doi:10.1172/jci.insight.133675)
Supplement: Supplemental data [file jciinsight-5-133675-s063.pdf]

Supplemental Section

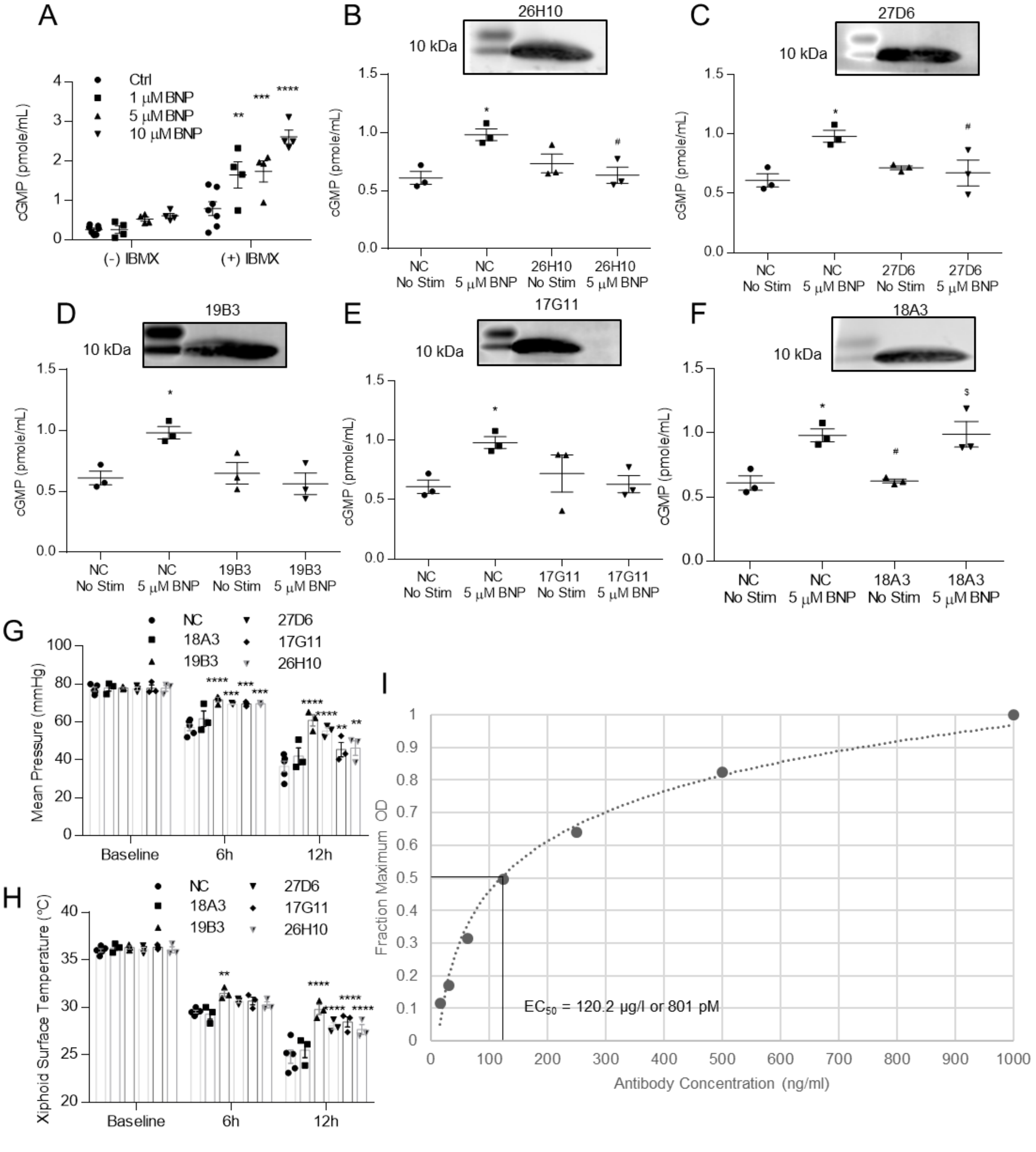

Supplemental Figure 1: Development of BNP Neutralizing Monoclonal Antibody. Screening of hybridoma supernatants with BNP-neutralizing activity in cultured HEK293 cells. **A:** cGMP levels in HEK293 cells treated with BNP with and without phosphodiesterase inhibitor IBMX. n=4-8 wells. **$p<0.01$ vs Ctrl, ***$p<0.001$ vs Ctrl, ****$p<0.0001$ vs Ctrl by Two-way ANOVA with Tukey multiple comparisons. **B-F:** Immunoblotting analysis of purified BNP-45 peptide run on SDS-PAGE using hybridoma supernatant as primary antibody, and cGMP levels in HEK293 cells co-treated with BNP

and supernatant from each of 5 different hybridoma clones. These clones include 26H10 (B), 27D6 (C), 19B3 (D), 17G11 (E), and 18A3 (F). NC stands for non-targeting control supernatant. n=3 wells/group. *$p<0.05$ vs NC No Stim, #$p<0.05$ vs NC 5μM BNP, \$$p<0.05$ vs 18A3 5μM BNP by one-way ANOVA with Tukey multiple comparisons. **G-H:** Mean arterial pressure (G) and surface temperature (H) of C57BL/6 mice that underwent CLP and were injected with hybridoma supernatant (~10-15 μg of antibody, I.P.) at the time of surgery. n=3-5 mice per group. **$p<0.01$, ***$p<0.001$, ****$p<0.0001$ vs NC. **I:** ELISA results provided by GenScript characterizing the affinity of purified antibody 19B3 to BNP antigen. Increasing concentrations of purified 19B3 was applied to wells coated with antigen to determine the EC_{50} of maximal binding.

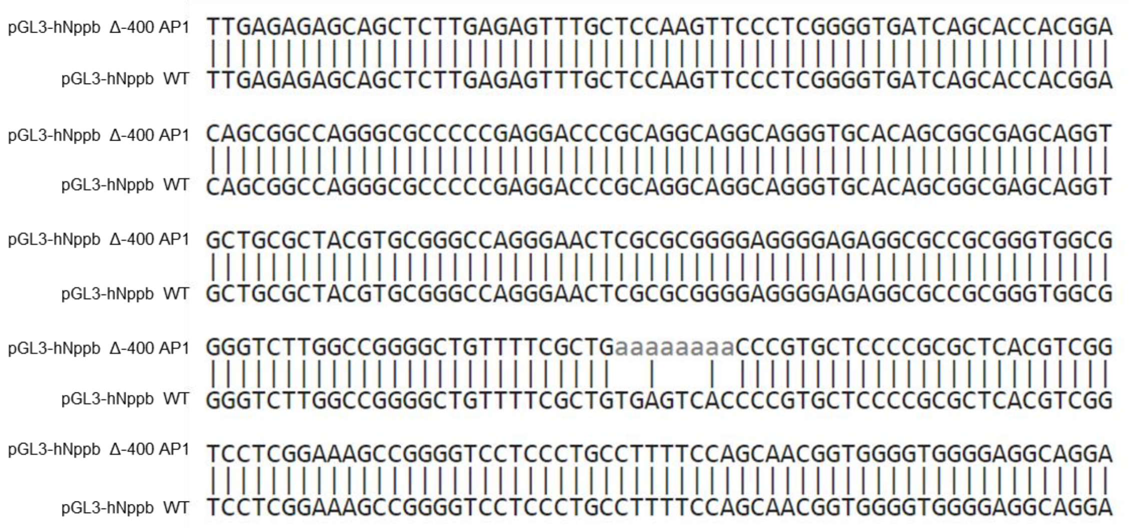

Supplemental Figure 2: Confirmation of Mutagenesis of the -400 AP1 Site. Comparison of the sequencing results of wild type and mutated human *Nppb* promoter fragment (-2000/+100 relative to the transcription start site) confirming mutation of the TGAGTCA AP1 site to AAAAAAA that was cloned in the KpnI and NheI restriction sites of the polylinker of the pGL3 vector (pGL3-hNppb Δ-400AP1).

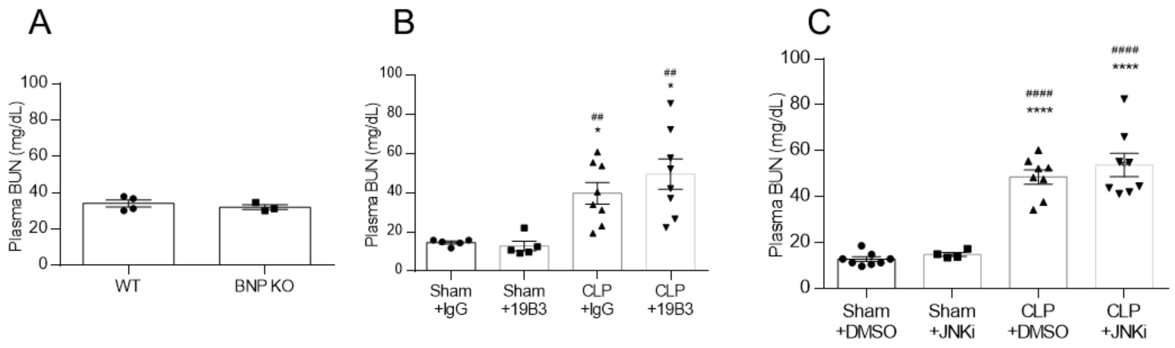

Supplemental Figure 3: BNP Inhibition and JNK Inhibition do not Affect Septic Kidney Injury. Plasma BUN in BNP KO and wild type control mice 12h post-CLP surgery. n=4 WT mice and n=3 BNP KO mice (A). No statistical significance by t-test. Plasma BUN in plasma collected from mice 12h post CLP and 6h post administration of IgG or 19B3 (B). n=5 Sham+IgG mice, n=5 Sham+19B3 mice, n=8 CLP+IgG mice, n=8 CLP+19B3 mice. *p<0.05 vs Sham+IgG, ##p<0.01 vs CLP+19B3 by One-Way ANOVA with Tukey multiple comparisons. Plasma BUN in plasma collected from mice 12h post CLP and 6h post administration of DMSO or JNKi (C). n=8 Sham+DMSO mice, n=4 Sham+JNKi mice, n=8 CLP+DMSO mice, n=8 CLP+JNKi mice. ****p<0.0001 vs Sham+DMSO, ####p<0.0001 vs Sham+JNKi by One-Way ANOVA with Tukey multiple comparisons.

		N	EDV (µl)	ESV (µl)	SV (µl)	CO (ml/min)	GLS (Peak %)	EF (%)	Heart Rate (bpm)	Temp (°C)	Weight (g)	Systolic (mmHg)	Diastolic (mmHg)	MAP (mmHg)	CO:BW (ml/min/g)
Baseline Measurements															
WT	Mean	4	42.30	15.93	26.43	13.93	-20.96	62.00	528.10	36.43	26.58	105.00	61.25	76.00	0.52
	STD		5.79	2.53	4.12	2.30		4.08	34.48	0.33	1.23	1.83	1.71	1.63	0.07
Nppb -/-	Mean	3	47.10	18.43	28.67	13.73	-20.74	60.67	477.60	36.27	25.60	106.00	62.00	76.33	0.53
	STD		3.96	3.92	2.95	2.35	0.72	6.81	40.95	0.40	1.01	3.61	1.00	0.58	0.08
6hr Measurements															
WT	Mean	4	24.55	11.13	13.43	6.50	-12.71	55.50	484.25	30.28	26.53	74.75	39.50	51.50	0.24
	STD		5.52	4.86	3.39	1.65	0.72	12.87	12.88	0.31	0.70	3.59	3.11	3.11	0.06
Nppb -/-	Mean	3	50.27	29.47	20.77	9.57	-8.92	41.33	427.00	31.87	24.91	92.33	57.67	69.67	0.38
	STD		2.59	2.75	0.71	1.29	1.87	2.52	14.33	0.32	1.07	4.51	5.03	2.52	0.06
12hr Measurements															
WT	Mean	4	16.73	5.85	10.83	4.65	-13.79	65.25	431.28	27.13	26.15	53.00	24.75	37.00	0.18
	STD		4.86	2.52	2.77	1.20	1.39	6.95	26.67	2.10	1.04	4.24	6.29	8.49	0.05
Nppb -/-	Mean	3	49.00	26.40	22.63	9.97	-9.83	46.00	438.27	30.63	24.57	75.33	42.00	54.33	0.41
	STD		8.46	5.72	3.25	1.72	1.23	3.61	14.17	0.40	1.39	2.08	4.00	3.06	0.06

Supplemental Table 1: Table of Values Obtained from 2D-Echo and Tail Cuff in BNP KO Mice. 2D-Echo echocardiography- and tail cuff cuff-derived measurements from wild type and *Nppb*$^{-/-}$ mice subjected to CLP or sham surgery.

		N	EDV (µl)	ESV (µl)	SV (µl)	CO (ml/min)	GLS (Peak %)	EF (%)	Heart Rate (bpm)	Temp (°C)	Weight (g)	Systolic (mmHg)	Diastolic (mmHg)	MAP (mmHg)	CO:BW (ml/min/g)
Baseline Measurements															
Sham +IgG	Mean	5	40.20	14.54	25.64	13.80	-21.04	63.60	537.86	35.82	25.30	115.00	57.80	77.00	0.55
	STD		5.37	2.76	2.93	1.59	1.97	2.70	19.39	0.42	4.24	3.24	3.27	2.45	0.08
Sham +19B3	Mean	5	40.50	11.66	28.84	14.94	-24.13	71.00	520.20	36.00	26.54	113.40	60.00	77.60	0.57
	STD		3.33	2.74	2.22	0.99	2.58	5.24	34.98	0.16	3.02	5.13	2.12	1.14	0.08
CLP +IgG	Mean	8	42.44	14.06	28.36	14.30	-22.79	67.00	504.75	35.89	23.05	112.00	59.38	76.75	0.63
	STD		6.79	3.40	3.96	2.03	1.76	3.78	32.23	0.32	1.80	3.51	1.77	2.05	0.13
CLP +19B3	Mean	8	41.49	15.13	25.86	13.21	-22.88	63.25	505.64	35.89	23.40	111.50	60.50	77.25	0.57
	STD		5.25	1.75	3.41	1.27	1.71	2.31	42.25	0.32	3.11	2.45	1.51	1.16	0.09
6hr Measurements															
Sham +IgG	Mean	5	41.94	16.04	25.92	13.24	-21.86	61.80	512.64	36.22	24.38	109.60	57.40	74.60	0.55
	STD		8.08	4.80	4.11	1.95	1.93	4.55	26.07	0.50	4.05	4.83	1.67	2.07	0.06
Sham +19B3	Mean	5	40.18	13.72	26.46	13.74	-21.53	65.40	517.36	36.20	25.86	110.40	58.60	75.60	0.53
	STD		8.17	2.34	5.92	3.50	1.68	1.82	30.38	0.16	2.84	4.72	1.82	2.41	0.09
CLP +IgG	Mean	8	15.26	5.21	10.05	4.74	-13.46	65.75	479.35	30.64	22.57	88.25	43.88	58.75	0.21
	STD		3.67	1.84	2.02	0.63	2.05	6.39	52.13	0.54	1.77	2.55	0.09	2.49	0.03
CLP +19B3	Mean	8	16.03	6.58	9.46	4.51	-14.24	59.38	479.60	30.73	22.69	87.00	43.75	57.88	0.20
	STD		2.36	2.15	1.36	0.69	1.30	9.36	39.09	0.51	3.07	1.51	2.55	1.81	0.04
12hr Measurements															
Sham +IgG	Mean	5	45.86	18.40	31.46	15.40	-21.12	68.00	493.92	35.94	24.02	111.40	55.20	74.20	0.65
	STD		3.59	4.89	4.67	1.24	1.19	6.82	38.58	0.18	4.02	5.32	2.39	2.68	0.09
Sham +19B3	Mean	5	40.10	12.94	27.16	14.00	-22.88	68.20	516.08	36.02	25.32	110.00	56.00	74.00	0.55
	STD		6.41	4.40	3.07	1.77	4.21	6.69	55.76	0.37	2.84	4.64	2.55	2.74	0.06
CLP +IgG	Mean	8	11.48	2.08	9.40	4.54	-12.76	82.00	485.23	26.83	22.05	65.63	25.63	38.88	0.21
	STD		1.96	1.26	1.94	0.89	1.91	10.56	32.95	1.56	1.83	10.29	6.46	7.32	0.05
CLP +19B3	Mean	8	26.05	11.53	14.55	6.90	-12.78	59.38	475.50	29.93	20.97	87.38	41.38	56.88	0.33
	STD		8.15	5.19	3.63	2.03	1.79	10.21	80.76	2.87	1.33	11.59	7.41	8.53	0.09

Supplemental Table 2: Table of Values Obtained from 2D-Echo and Tail Cuff in Mice Treated with 19B3. 2D-Echo echocardiography- and tail cuff cuff-derived measurements from C57BL/6 mice subjected to CLP or sham surgery and treated with 19B3 or mouse IgG (2mg/kg) 6h post-surgery.

		N	EDV (µl)	ESV (µl)	SV (µl)	CO (ml/min)	GLS (Peak %)	EF (%)	Heart Rate (bpm)	Temp (°C)	Weight (g)	Systolic (mmHg)	Diastolic (mmHg)	MAP (mmHg)	CO:BW (ml/min/g)
Baseline Measurements															
Sham + DMSO	Mean	8	41.44	12.71	28.70	14.44	-21.15	69.00	498.36	36.65	27.66	109.88	62.13	77.50	0.52
	STD		3.35	4.05	3.75	3.11	4.5	8.86	59.77	0.48	1.73	5.44	3.36	2.39	0.11
Sham + JNKi	Mean	4	46.28	14.85	30.60	16.55	-23.21	67.50	518.65	36.28	27.95	109.00	60.25	75.50	0.60
	STD		4.22	2.09	6.23	3.85	4.27	4.73	23.54	0.38	2.54	6.63	1.26	3.87	0.18
CLP+ DMSO	Mean	8	46.51	14.46	32.06	16.69	-22.79	70.00	523.96	36.50	29.49	106.38	63.13	77.38	0.57
	STD		11.21	6.78	5.91	3.11	2.37	8.55	56.67	0.37	3.52	5.45	5.57	4.41	0.13
CLP+ JNKi	Mean	8	47.84	16.75	31.06	15.40	-21.88	65.25	502.94	36.20	29.45	107.38	61.88	79.00	0.54
	STD		10.67	5.86	6.03	2.27	2.59	6.32	60.31	0.27	4.06	5.76	4.88	3.12	0.13
6hr Measurements															
Sham + DMSO	Mean	8	45.16	16.61	29.51	15.63	-21.82	64.50	530.14	36.20	26.70	109.13	62.13	74.38	0.59
	STD		11.32	7.96	3.90	2.36	3.09	7.80	44.38	0.94	2.12	5.51	7.77	5.48	0.09
Sham + JNKi	Mean	4	46.65	20.13	27.48	13.43	-21.74	56.75	487.75	36.40	27.23	106.00	61.25	76.25	0.50
	STD		6.39	4.37	2.66	1.77	1.98	4.35	39.67	0.22	2.54	5.16	4.27	4.11	0.07
CLP+ DMSO	Mean	8	18.73	10.86	7.84	3.88	-8.91	46.25	535.31	30.33	28.76	87.38	49.25	61.88	0.14
	STD		6.67	6.63	4.67	2.45	4.90	27.54	191.63	0.55	3.52	5.37	4.77	4.70	0.09
CLP+ JNKi	Mean	8	21.53	13.41	8.13	3.93	-8.78	37.63	494.51	30.28	28.60	86.75	49.75	62.13	0.14
	STD		7.36	5.65	4.28	1.90	3.26	15.71	65.99	0.37	3.86	5.99	5.12	5.06	0.07
12hr Measurements															
Sham + DMSO	Mean	8	47.33	18.10	30.03	15.58	-22.41	63.00	509.21	35.95	25.61	105.13	61.13	75.75	0.61
	STD		14.42	8.51	5.89	3.76	2.82	5.76	45.93	0.66	1.43	2.59	2.17	2.25	0.15
Sham + JNKi	Mean	4	42.53	13.55	30.75	14.85	-23.81	68.00	503.65	36.25	26.13	103.00	63.50	76.75	0.57
	STD		4.21	3.46	5.36	2.10	2.28	4.97	80.63	0.25	2.64	2.71	1.91	2.22	0.09
CLP+ DMSO	Mean	8	8.73	4.61	4.10	1.75	-6.27	59.13	417.41	25.85	28.29	62.88	28.25	48.88	0.06
	STD		6.91	5.21	2.53	1.18	2.95	24.82	48.83	1.57	3.51	9.72	7.40	13.81	0.05
CLP+ JNKi	Mean	8	32.69	16.83	15.86	6.56	-14.34	50.00	420.74	28.96	28.03	95.38	56.75	69.50	0.24
	STD		20.51	12.18	9.74	3.98	4.77	13.56	43.74	1.69	3.99	4.21	5.70	5.15	0.15

Supplemental Table 3: Table of Values Obtained from 2D-Echo and Tail Cuff in Mice Treated with JNK inhibitor. 2D-echocardiography- and tail cuff-derived measurements from mice subjected to CLP or sham surgery and treated with SP600125 (JNKi, 5mg/kg) or DMSO 6h post-surgery.

Gene	Application	Species	Fwd Primer	Rev Primer
BNP	rtPCR	*Mus musculus*	5’-ggaggtcactcccatcctct-3’	5’-agctgtctctgggccatttc-3’
36B4	rtPCR	*Mus musculus*	5’-gcgacctggaagtccaactac-3’	5’-atctgctgcatctgcttgg-3’
BNP	rtPCR	*Homo sapiens*	5’-gctcctgctcttcttgcatc-3’	5’-ggacttccagacacctgtgg-3’
18s rRNA	rtPCR	*Homo sapiens*	5’-gagccacccgagattgagca-3’	5’-tagtagcgacgggcggtgtg-3’
-400 AP1 ChIP	ChIP	*Homo sapiens*	5’-gttttcgctgtgagtcac-3’	5’-cgttgctggaaaaggcag-3’
BNP Promoter (-2000bp)	Cloning	*Homo sapiens*	5’-aaaaaaggtaccccagcactggtgtctttaca-3’	5’-aaaaaagctagcgtctctggagggactgcgga-3’
-400 AP1 Mutation	Site Directed Mutagenesis	*Homo sapiens*	5’-aaaacccgtgctccccgcgctc-3’	5’-ttttcagcgaaaacagccccggcc-3’

Supplemental Table 4: Primer Sequences. Primers used for rtPCR of indicated genes, ChIP qPCR of the *Nppb* promoter, and cloning/mutagenesis of the *Nppb* promoter sequence.
